# ZnCo_2_O_4_@g-C_3_N_4_@Cu as a new and highly efficient heterogeneous photocatalyst for visible light-induced cyanation and Mizoroki–Heck cross-coupling reactions†

**DOI:** 10.1039/d4ra04827j

**Published:** 2024-08-22

**Authors:** Roya Jahanshahi, Hadis Hosseini Moghadam, Sara Sobhani, José Miguel Sansano

**Affiliations:** a Department of Chemistry, College of Sciences, Shiraz University Shiraz 71454 Iran; b Department of Chemistry, College of Sciences, University of Birjand Birjand Iran ssobhani@birjand.ac.ir; c Departamento de Química Orgánica, Facultad de Ciencias, Centro de Innovación en Química Avanzada (ORFEO-CINQA), Instituto de Síntesis Orgánica (ISO), Universidad de Alicante Apdo. 99 03080-Alicante Spain

## Abstract

Conducting C–C cross-coupling reactions under convenient and mild conditions remains extremely challenging in traditional organic synthesis. In this study, ZnCo_2_O_4_@g-C_3_N_4_@Cu exhibited extraordinary photocatalytic performance as a new visible light harvesting heterogeneous copper-based photocatalyst in cyanation and Mizoroki–Heck visible-light-driven cross-coupling reactions at room temperature and in air. Surprisingly, by this method, the cyanation and Mizoroki–Heck cross-coupling reactions of various iodo-, bromo- and also the challenging chloroarenes with respectively K_4_[Fe(CN)_6_]·3H_2_O and olefins produced promising results in a sustainable and mild media. The significant photocatalytic performance of ZnCo_2_O_4_@g-C_3_N_4_@Cu arises from the synergistic optical properties of ZnCo_2_O_4_, g-C_3_N_4_, and Cu. These components can enhance the charge carrier generation and considerably reduce the recombination rate of photogenerated electron–hole pairs. No need to use heat or additives, applying an economical and benign light source, utilizing an environmentally compatible solvent, facile and low-cost photocatalytic approach, aerial conditions, high stability and convenient recyclability of the photocatalyst are the remarkable highlights of this methodology. Moreover, this platform exhibited the ability to be performed on a large scale, which is considered an important issue in industrial and pharmaceutical use. It is worth noting that this is the first time that a heterogeneous copper-based photocatalyst has been employed in visible light-promoted cyanation reactions of aryl halides.

## Abstract 2

No need to heat or additives, applying an economical and benign light source, utilizing an environmentally compatible solvent, aerial conditions, high stability and convenient recyclability of the catalyst are the remarkable highlights of this method.

## Graphical abstract

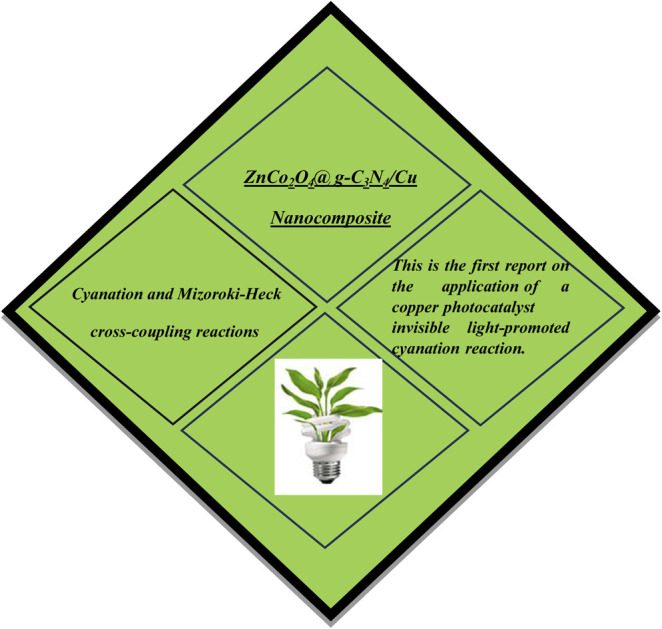

## Introduction

Nowadays, enhanced environmental consciousness has promoted the efficiency of the chemical processes under more benign and sustainable conditions, both in industrial and academic contexts. The main factors supporting chemical transformations toward green chemistry are using eco-friendly heterogeneous catalysts, safe and benign solvents and alternative energy sources.^1^ In this context, the establishment of visible light harvesting-based photocatalysis has offered a technically attractive and energy-saving platform to effectually promote the chemical processes under mild conditions.^2^ The use of visible light mediated strategies is highly recommended, since it is clean, abundant in the solar spectrum (44%), easy to access and accompanied with less side-reactions.^3,4^

Graphitic carbon nitride (g-C_3_N_4_) is a non-metallic semiconductor with photo-responsive attributes, making it a precious candidate for environmental remediation.^5,6^ Due to having the advantages of easy preparation, narrow band gap energy (2.73 eV), low-cost, chemical/thermal robustness and nontoxicity, it has been extensively investigated for visible light mediated photocatalytic processes.^7^ However, the rapid recombination of photo-excited electrons/holes in g-C_3_N_4_ has limited its efficient photocatalytic performance.^7^ Incorporation of g-C_3_N_4_ with other metal oxide semiconductors could effectually improve its visible light photocatalytic properties.^6^

As an affordable, environmentally friendly, and capable transition bimetallic oxide, ZnCo_2_O_4_ has been reported to be a supreme candidate to make effective heterojunctions with other semiconductors.^8,9^ ZnCo_2_O_4_ with the band gap energy of about 2.32 eV has specific optoelectronic features, resulting to decrease the rate of photogenerated electron/hole pairs recombination.^9^ Moreover, owing to the particular crystalline lattice and the synergistic effect of two metal species, ZnCo_2_O_4_ shows superior photoelectrochemical stability and electron conductivity in comparison with single metal oxides such as Co_3_O_4_ and ZnO.^10,11^ Inspired by this consideration, it is supposed that the band edge of ZnCo_2_O_4_ could match appropriately with g-C_3_N_4_ one. So, the constructed heterojunction composed of both semiconductors have the ability of enhancing the visible light absorption potential of the final composite by slowing down the recombination velocity of the photo-excited electron–hole pairs.^8^

Recent studies have revealed that incorporating noble metal nanoparticles (NPs) into photocatalytic structures can substantially boost the photocatalytic performance of the photocatalyst, particularly when exposed to the visible light irradiation.^12–14^ Amongst them, copper NPs with a plasmonic nanostructure^15–18^ have attracted increasing attention in photocatalytic developments owing to the stability, non-toxicity, low-cost, and availability.^19,20^ By the introduction of copper NPs to the photocatalyst structure, the appropriate separation of charge carriers would efficiently increase, which can prominently improve the visible-light harvesting potential of the photocatalytic system.^12–14^

**Fig. 1:**
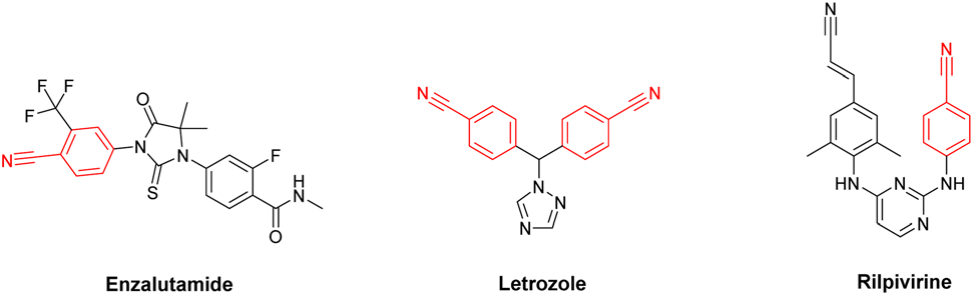
Structures of some drugs containing aryl nitrile moiety.

Cyanation reaction of aryl halides can be considered as one of the most synthetically important reactions, since the resulting aryl nitriles constitute an important class of organic intermediates with widespread applications as pharmaceuticals, herbicides, perfumes, polymers, dyes and natural products.^15–20^ For instance, the Enzalutamide (anti-prostate cancer), Letrozole (anti-breast cancer) and Rilpivirine (anti-HIV), which were amongst the top 200 brand-name drugs by retail sales in 2019,^21^ possess nitrile motif in their composition (Fig. 1).

The conventional known approaches for the synthesis of aryl nitriles are consist of the Rosenmund-von Braun and Sandmeyer reactions.^22,23^ These procedures suffered from critical complications, such as the elevated reaction temperatures and needing the stoichiometric quantities of extremely toxic CuCN. Transition metal-catalyzed cyanation of aryl halides has evolved rapidly as an effectual alternative for the preparation of aryl nitriles in rather milder and benign conditions.^24,25^ Along this line, Pd as the most widely used transition-metal in cross-coupling reactions,^26–28^ and a few examples of Ni,^29^ Co,^30^ Ir,^31^ Rh,^32^ and Cu based^33^ catalytic systems with different cyanide reagents such as KCN,^34^ CuCN,^35^ NaCN,^36^ CuSCN,^37^ Zn(CN)_2_,^38^ AgCN,^39^ TMSCN,^40^*etc.*, have been developed to perform the cyanation reactions. However, the high toxicity or sensitivity to the moisture of most cyanide sources seriously limited the industrial applications of these protocols. Currently, K_4_[Fe(CN)_6_]·3H_2_O has become as the cyanide reagent of choice in cyanation reaction of different aryl halides. Compared with the conventional cyanide sources, K_4_[Fe(CN)_6_]·3H_2_O is non-toxic, non-hygroscopic, convenient to use, cheap and commercially available. As an important issue, the predomination on severe affinity of metal catalysts for cyanide groups is a challenging problem that should be seriously considered in the cyanation reactions. Interestingly, the gradual release of cyanide ions from K_4_[Fe(CN)_6_]·3H_2_O could effectually address this problem and hinder the catalyst deactivation.

There are a limited number of copper-catalyzed cyanation reactions of aryl halides with different cyanide sources.^41–51^ Among them, a few reports are related to the use of K_4_[Fe(CN)_6_]·3H_2_O.^46–51^ These procedures usually required harsh reaction conditions, rather expensive/toxic additives or ligands, hazardous solvents, prolonged reaction times and are associated with complicated product isolation and unsatisfied yields. A literature survey clearly pointed out that there is only one report for the light-assisted cyanation reaction of aryl halides using a copper-based photocatalyst.^52^ This procedure was accomplished using NaCN as a cyanide source. It has been performed under harmful UV-light irradiation and required very strict reaction conditions, alongside a complicated catalyst. Surprisingly, there is not any report on the visible light-promoted cyanation reactions of aryl halides catalyzed by a heterogeneous copper-based photocatalyst.

**Fig. 2:**
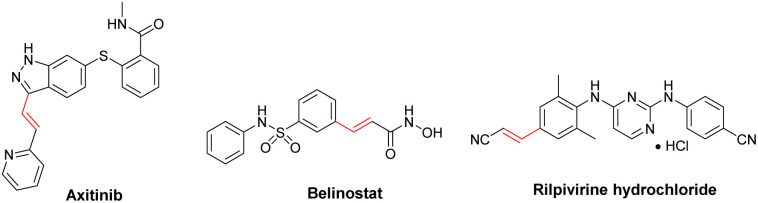
Structures of some drugs prepared by Mizoroki–Heck cross-coupling reaction.

Mizoroki–Heck cross-coupling reaction of aryl halides with olefins is one of the most crucial routes for the carbon–carbon bond generation in synthetic organic chemistry. The resulting coupling products are fundamental units in the synthesis of bioactive compounds, polymers, natural products and pharmaceuticals.^53–58^Fig. 2 shows the structures of some drugs prepared by Mizoroki–Heck cross-coupling reaction.

Notably, most of the methodologies developed for the Mizoroki–Heck reactions rely on using Pd-based catalysts, costly/toxic solvents or bases, elevated temperatures and harsh reaction conditions. Although some of these issues can be mitigated by conducting the reactions under light irradiation, there are only a few reports on visible light-induced Mizoroki–Heck cross-coupling reactions.^59–65^ Unfortunately, most of these methods remain unaffordable and involve strict and complex photocatalytic reaction conditions. Hence, the introduction of a cost-effective heterogeneous photocatalyst to proceed the Mizoroki–Heck cross-coupling reactions through a mild, efficient and environmentally compatible procedure has received significant interest.

In continuation of our research interest to establish the new and efficient catalytic systems for sustainable and benign progress of cross-coupling reactions,^66–72^ herein, we have introduced ZnCo_2_O_4_@g-C_3_N_4_@Cu^73^ as a new and proficient visible light-induced photocatalyst for conducting the cyanation and Mizoroki–Heck reactions using visible light irradiation at room temperature and in air atmosphere.

## Results and discussion

### Synthesis and characterization of ZnCo_2_O_4_@g-C_3_N_4_@Cu

**Scheme 1:**
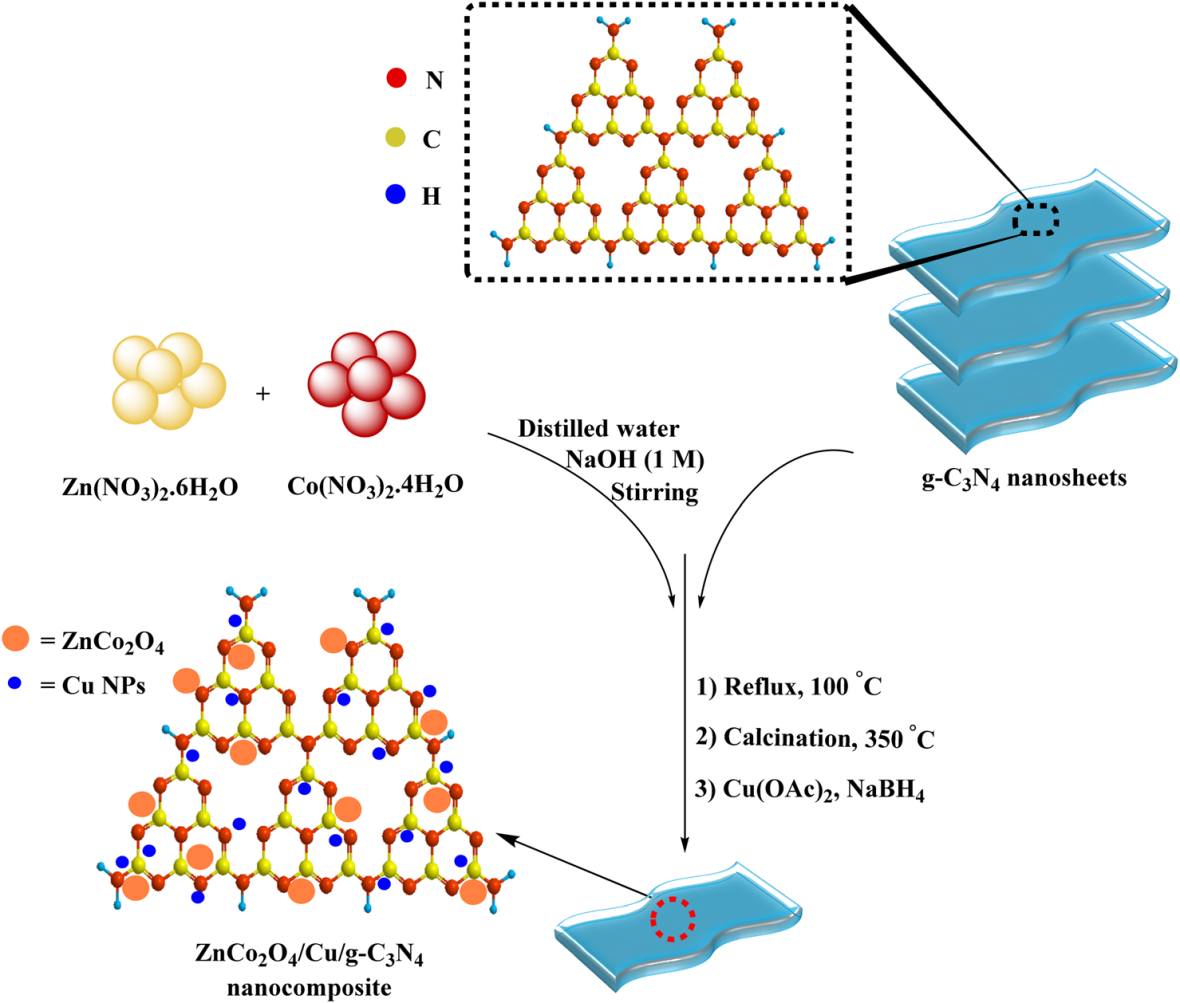
Schematic representation for the preparation of ZnCo_2_O_4_@g-C_3_N_4_@Cu.

The photocatalyst was prepared based on the process shown in Scheme 1. Initially, g-C_3_N_4_ was synthesized by sequential polymerization and liquid exfoliation techniques. Subsequently, the resulting g-C_3_N_4_ was added to an alkaline mixture of Co(NO_3_)_2_·6H_2_O and Zn(NO_3_)_2_·6H_2_O, stirring under reflux. Heating the obtained raw ZnCo_2_O_4_@g-C_3_N_4_ sample at 350 °C, followed by reducing Cu(OAc)_2_ on the surface of ultimate ZnCo_2_O_4_@g-C_3_N_4_ afforded the desired ZnCo_2_O_4_@g-C_3_N_4_@Cu. The freshly synthesized ZnCo_2_O_4_@g-C_3_N_4_@Cu was characterized by different techniques.

### FT-IR spectra

FT-IR spectra of the pristine g-C_3_N_4_ and ZnCo_2_O_4_@g-C_3_N_4_@Cu are presented in Fig. S1.† In the FT-IR spectrum of g-C_3_N_4_ (Fig. S1a†), the indicative intense adsorption band at 808 cm^−1^ could be certified to the breathing mode of triazine units. The board adsorption bands located at 1200–1650 cm^−1^ could be ascribed to the stretching vibrations of C–N and C=N bonds. In addition, the broad adsorption band at about 3000–3400 cm^−1^ could be allocated to the stretching vibration modes of –NH bonds and the adsorbed water molecules. As it is obvious, the characteristic bands of g-C_3_N_4_ could be easily detected in the FT-IR spectrum of ZnCo_2_O_4_@g-C_3_N_4_@Cu (Fig. S1b†). Besides, the appearance of a new distinct absorption band at 638 cm^−1^ is consigned to the stretching modes of Co–O bonds in the catalyst structure.

### EDS analysis and elemental mapping images

EDS analysis of the ZnCo_2_O_4_@g-C_3_N_4_@Cu demonstrated the presence of O, N, C, Zn, Co and Cu elements (Fig. S2a†). Existence of these elements with homogeneous distribution on the entire surface of the catalyst in the elemental mapping images (Fig. S2b–h†), showed the uniformity of the elemental composition of ZnCo_2_O_4_@g-C_3_N_4_@Cu.

### ICP-OES analysis

ICP-OES analysis was determined the copper content of the photocatalyst and showed that 1 g of ZnCo_2_O_4_@g-C_3_N_4_@Cu contains 0.53 mmol of Cu.

### FESEM and TEM analyses

**Fig. 3:**
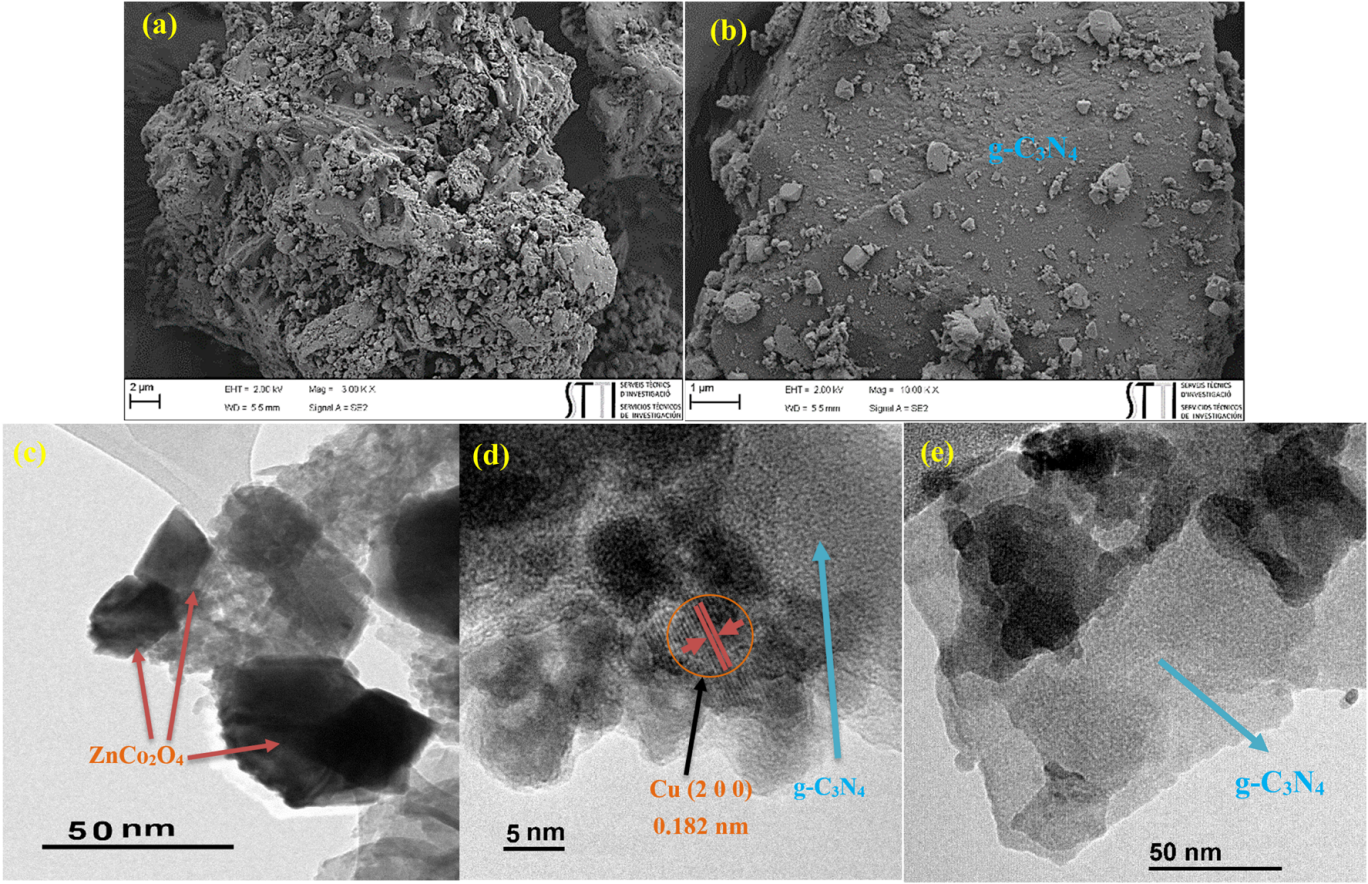
(a and b) FESEM images of ZnCo_2_O_4_@g-C_3_N_4_@Cu, and (c–e) TEM images of ZnCo_2_O_4_@g-C_3_N_4_@Cu.

The morphology of the photocatalyst was probed by FESEM and TEM analyses (Fig. 3). As it is evident from the images, g-C_3_N_4_ sheet-like structure accompanied with the cubic ZnCo_2_O_4_ can be recognized. The average size of ZnCo_2_O_4_ was calculated to be in the range of 25 to 40 nm (Fig. 3c). Furthermore, Fig. 3d showed the lattice fringe at about 0.182 nm, which was consistent with (2 0 0) planes in copper NPs. The average particle sizes of Cu NPs were measured to be 3–5 nm (Fig. 3d).

### XPS analysis

XPS analysis was performed to probe the electronic properties and elemental composition of ZnCo_2_O_4_@g-C_3_N_4_@Cu (Fig. S3†). In the XPS plot of the photocatalyst (Fig. S3a†), distinct signals attributed to C, O, N, Co, Zn, and Cu elements are clearly detected. In the high-resolution XPS spectrum of C 1 s, the appearance of peaks at 284.5, 285.8, 287.6 and 288.8 eV are assigned to the adventitious C–C bonds, sp^2^ carbons in the C–N–C, N=C–(N)_2_, and N=C(N)–NH_2_ and N=C(N)–NH, respectively (Fig. S3b†). In Fig. S3c,† the high-resolution XPS spectra of O 1s was represented three peaks at 530.4, 531.6 (which are both correspond to the crystal lattice oxygen atoms) and 533.2 eV (which indexed to the adsorbed O_2_ and H_2_O molecules on the catalyst surface). Fig. S3d† indicates the high-resolution XPS spectrum of N 1s. In this spectrum, the presence of three peaks at 400.7, 398.3, and 399.4 eV refers to the amino groups (C–NH_*x*_), sp^2^ nitrogen atoms (C–N=C), and sp^3^ nitrogen atoms (H–N–C_3_), respectively. The characteristics of Zn 2p_3/2_ and Zn 2p_1/2_ in Zn^2+^, arise from the signal peaks at 1021.0 and 1043.9 eV, respectively (Fig. S3e†). As it is evident in Fig. S3f,† the indicative peaks at 779.8 eV (Co 2p_3/2_) and 795.0 eV (Co 2p_1/2_) are ascribed to Co^3+^, and peaks at 781.9 eV (Co 2p_3/2_) and 796.6 eV (Co 2p_1/2_) are identified as the Co^2+^ species. Two other peaks at 785.3 and 800.0 are assigned to the shake-up satellites, further elucidating the presence of multivalent cobalt. The typical characteristics of Cu in a zero-oxidation state were verified through the observation of binding energies at 932.5 eV (Cu 2p_3/2_) and 952.2 eV (Cu 2p_1/2_) (Fig. S3g†). The indicative peaks of Cu 2p_3/2_ positioned at 934.3 and the peaks of Cu 2p_1/2_ at 954.0 are related to Cu^1+^ in the composite. The two minor peaks located at 936.3 and 955.7 eV are ascribed to Cu(ii) 2p_3/2_ and Cu(ii) 2p_1/2_, respectively. Moreover, two little satellite peaks are presented at 940.8 and 943.5 eV.

### XRD analysis

To elucidate the structural properties of the synthesized photocatalyst, XRD analysis of ZnCo_2_O_4_@g-C_3_N_4_ and ZnCo_2_O_4_@g-C_3_N_4_@Cu was conducted (Fig. S4†). Fig. S4 (a and b†) depicted the indicative diffraction peaks at 13.1° and 27.3°, which are correspond to (1 0 0) and (0 0 2) crystal planes of g-C_3_N_4_ (JCPDS 87-1526),^74^ respectively. The diffraction signals at 31.2°, 36.6°, 44.7°, 59.2° and 65.1° (2*θ*) related, respectively, to (2 2 0), (3 1 1), (4 0 0), (5 1 1) and (4 4 0) crystal planes in ZnCo_2_O_4_ were well in accordance with the cubic spinel structure (JCPDS 23-1390)^75^ (Figure S4 a and b†). The appearance of peaks at 2*θ*° = 43.3°, 50.4°, 74.1° in the XRD pattern of ZnCo_2_O_4_@g-C_3_N_4_@Cu (Fig. S4b†), which were indexed to the reflection planes (1 1 1), (2 0 0) and (2 2 0), respectively, certified the presence of Cu NPs in the composite (JCPDS 04-0836).^76^

### UV-vis DRS and Tauc plot

**Fig. 4:**
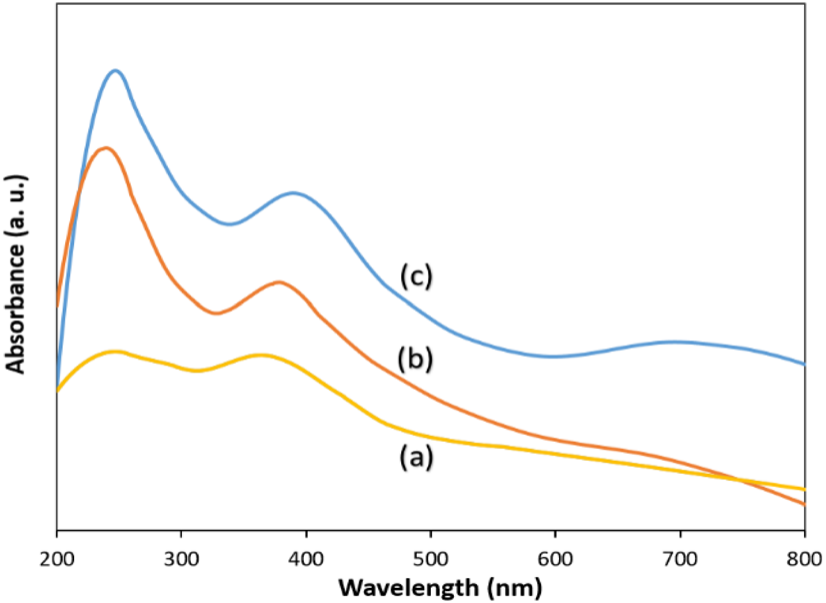
UV-vis DRS of (a) g-C_3_N_4_, (b) ZnCo_2_O_4_@g-C_3_N_4_ and (c) ZnCo_2_O_4_@g-C_3_N_4_@Cu.

The optical absorption properties of g-C_3_N_4_, ZnCo_2_O_4_@g-C_3_N_4_ and ZnCo_2_O_4_@g-C_3_N_4_@Cu were investigated by UV-vis diffuse reflectance spectroscopy (DRS) technique (Fig. 4).

Compared with the pure g-C_3_N_4_, which has good absorption in the visible light area (Fig. 4a), ZnCo_2_O_4_@g-C_3_N_4_ showed enhanced absorptive capacity in the visible light region (Fig. 4b), which originates from the promoted separation rate of the photogenerated charges.^77,78^ Incorporation of Cu to the composite could effectually improve the optical capability of the photocatalyst, resulted in an intense absorption of ZnCo_2_O_4_@g-C_3_N_4_@Cu in the visible light region (Fig. 4c), which can be attributed to an increase in the multiple internal scattering of light due to the presence of copper NPs.^79,80^ Based on the Tauc plot of (*αhν*)^2^*vs. hν* (Fig. S5†), band gap energy for ZnCo_2_O_4_@g-C_3_N_4_@Cu was estimated to be 2.3 eV. These results confirm the high ability of the photocatalyst for enhancing the separation efficiency of the photo-induced electron–hole pairs and so improving the visible-light photocatalytic performance.

### C–C cross-coupling reactions over ZnCo_2_O_4_@g-C_3_N_4_@Cu as a heterogeneous Cu-based photocatalyst

#### Arylcyanation reaction of aryl halides

To probe the catalytic activity of ZnCo_2_O_4_@g-C_3_N_4_@Cu for the cyanation reactions, the reaction of iodobenzene with K_4_[Fe(CN)_6_]·3H_2_O was selected as a typical reaction and the effect of several parameters such as solvents, bases, the catalyst amounts, and light sources was investigated towards the progression of the reaction (Table 1, entries 1–23). The obtained results clearly showed that the superior yield of the desired product was achieved in H_2_O : EtOH (1 : 1), Et_3_N and white LED lamp (20 W) by using 0.7 mol% of the photocatalyst, as the optimum reaction conditions (Table 1, entry 14). Separately studied control experiments under dark reaction conditions and in the absence of the catalyst, did not lead to the desired product (Table 1, entries 24 and 25). Likewise, only a trace amount of the product was acquired in the absence of the base, after 24 h (Table 1, entry 26). Next, the catalytic potential of g-C_3_N_4_, Cu(OAc)_2_ and ZnCo_2_O_4_@g-C_3_N_4_ were individually evaluated in the model reaction at the same conditions (Table 1, entries 27–29). No product was attained by using g-C_3_N_4_ (Table 1, entry 27) and trace amount of the desired product was acquired over Cu(OAc)_2_ (Table 1, entry 28). A similar reaction by applying ZnCo_2_O_4_@g-C_3_N_4_ was associated with a poor yield of the product even after 24 h (Table 1, entry 29). These observations clearly showed that the boosted photocatalytic performance of ZnCo_2_O_4_@g-C_3_N_4_@Cu may be related to collaborative influence of the photocatalyst components. It is assumed that the visible light harvesting capability of the photocatalyst is directly refers to the appropriate combination of all photocatalytic partners, which can facilitate the electron conductivity in ZnCo_2_O_4_@g-C_3_N_4_@Cu. This phenomenon favors the efficient charge separation and prolongs the lifetime of photo-induced electrons and holes. Interestingly, as the electron reservoir species, Cu NPs can trap the photo-promoted electrons and speed up the reaction under the visible light illumination.

**Table 1:** Screening the reaction conditions for the visible light-induced cyanation reaction of iodobenzene with K_4_[Fe(CN)_6_]·3H_2_O over ZnCo_2_O_4_@g-C_3_N_4_@Cu

Entry	Catalyst amount^a^ (mol%)	Solvent	Base	Light source	Time (h)	Isolated yield^b^ (%)
1	0.5	H_2_O : EtOH (1 : 1)	Et_3_N	White LED (20 W)	4/10	63/63
2	0.5	DMF	Et_3_N	White LED (20 W)	9	45
3	0.5	EtOH	Et_3_N	White LED (20 W)	24	56
4	0.5	H_2_O	Et_3_N	White LED (20 W)	24	30
5	0.5	CH_3_CN	Et_3_N	White LED (20 W)	24	—
6	0.5	EtOAc	Et_3_N	White LED (20 W)	24	Trace
7	0.5	HOAc	Et_3_N	White LED (20 W)	4	25
8	0.5	Toluene	Et_3_N	White LED (20 W)	24	—
9	0.5	H_2_O : EtOH (1 : 1)	NaOH	White LED (20 W)	24	34
10	0.5	H_2_O : EtOH (1 : 1)	K_2_CO_3_	White LED (20 W)	6	59
11	0.5	H_2_O : EtOH (1 : 1)	K_3_PO_4_	White LED (20 W)	5	47
12	0.4	H_2_O : EtOH (1 : 1)	Et_3_N	White LED (20 W)	8	50
13	0.6	H_2_O : EtOH (1 : 1)	Et_3_N	White LED (20 W)	7	72
**14**	**0.7**	**H**_**2**_ **O : EtOH (1 : 1)**	**Et**_**3**_ **N**	**White LED (20 W)**	**7**	**91**
15	0.75	H_2_O : EtOH (1 : 1)	Et_3_N	White LED (20 W)	7	91
16	0.8	H_2_O : EtOH (1 : 1)	Et_3_N	White LED (20 W)	8	82
17	0.7	H_2_O : EtOH (1 : 1)	Et_3_N	Blue LED (20 W)	10	40
18	0.7	H_2_O : EtOH (1 : 1)	Et_3_N	Green LED (20 W)	7	62
19	0.7	H_2_O : EtOH (1 : 1)	Et_3_N	Yellow LED (40 w)	9	57
20	0.7	H_2_O : EtOH (1 : 1)	Et_3_N	Sunlight^c^	8	60
21	0.7	H_2_O : EtOH (1 : 1)	Et_3_N	White LED (10 W)	7	43
22	0.7	H_2_O : EtOH (1 : 1)	Et_3_N	White LED (15 W)	7	68
23	0.7	H_2_O : EtOH (1 : 1)	Et_3_N	White LED (40 W)	7	91
24	0.7	H_2_O : EtOH (1 : 1)	Et_3_N	Dark	24	—
25	0	H_2_O : EtOH (1 : 1)	Et_3_N	White LED (20 W)	24	—
26	0.7	H_2_O : EtOH (1 : 1)	—	White LED (20 W)	24	Trace
27^d^	0.7	H_2_O : EtOH (1 : 1)	Et_3_N	White LED (20 W)	24	—
28^e^	0.7	H_2_O : EtOH (1 : 1)	Et_3_N	White LED (20 W)	24	Trace
29^f^	0.7	H_2_O : EtOH (1 : 1)	Et_3_N	White LED (20 W)	24	30

a Based on copper content (except for entries 25, 27 and 29).

b Reaction conditions: iodobenzene (1 mmol), base (1 mmol), K_4_[Fe(CN)_6_]·3H_2_O (0.4 mmol), solvent (4 mL), and ZnCo_2_O_4_@g-C_3_N_4_@Cu (except for entries 25 and 27–29), under visible light. During all experiments, to prevent photothermal side reactions, the reaction vessel was immersed in a water bath maintained at 25 °C.

c This experiment was accomplished in Birjand city (Iran), in a summer day, from 9:00 a.m. to 15:00 p.m. at 25 °C.

d Reaction was implemented using g-C_3_N_4_ as the catalyst.

e Reaction was implemented using Cu(OAc)_2_ as the catalyst.

f Reaction was implemented using ZnCo_2_O_4_@g-C_3_N_4_ as the catalyst.

The scope of cyanation cross-coupling reaction over ZnCo_2_O_4_@g-C_3_N_4_@Cu was extended to various aryl halides with K_4_[Fe(CN)_6_]·3H_2_O under optimal conditions (Table 2). As illustrated in Table 2, divers iodoarenes (Table 2, entries 1–4), bromoarenes (Table 2, entries 5–12) and chloroarenes (Table 2, entries 13–17) as the most challenging coupling partners, with easier availability and increased affordability compared to the aryl iodides and aryl bromides, participated in the cyanation cross-coupling reaction with K_4_[Fe(CN)_6_]·3H_2_O to give the desired aryl nitriles in good to high yields. In the cases of 1,4-diiodobenzene and 1,4-dibromobenzene, 0.8 mmol of K_4_[Fe(CN)_6_]·3H_2_O was used as the cyanating agent and 1,4-dicyanobenzene was generated (Table 2, entries 3 and 12). It is worth mentioning that in all cases, the reactions were clean and no homocoupling product was detected.

**Table 2:** Substrate scope for the cyanation reaction over ZnCo_2_O_4_@g-C_3_N_4_@Cu under visible light illumination

Entry	Ar	X	Time (h)	Isolated yield^a^ (%)
1	Ph	I	7	91
2	4-I-C_6_H_4_	I	14	76
3^b^	4-I-C_6_H_4_	I	15	87
4	4-MeO-C_6_H_4_	I	10	75
5	Ph	Br	8	90
6	4-O_2_N-C_6_H_4_	Br	7	86
7	4-NC-C_6_H_4_	Br	7	82
8	4-Me-C_6_H_4_	Br	12	70
9	4-MeO-C_6_H_4_	Br	12	76
10	4-F-C_6_H_4_	Br	16	73
11	3-Pyridyl	Br	10	92
12^b^	4-Br-C_6_H_4_	Br	14	71
13	Ph	Cl	12	88
14	4-O_2_N-C_6_H_4_	Cl	11	80
15	4-NC-C_6_H_4_	Cl	11	78
16	4-Me-C_6_H_4_	Cl	16	65
17	4-OHC-C_6_H_4_	Cl	14	85

a Reaction conditions: aryl halide (1 mmol), K_4_[Fe(CN)_6_]·3H_2_O (0.4 mmol), Et_3_N (1 mmol), ZnCo_2_O_4_@g-C_3_N_4_@Cu (0.7 mol%) and H_2_O : EtOH (1 : 1, 4 mL), under white LED lamp (20 W). To prevent any photothermal effect, the reaction vessel was immersed in a water bath maintained at 25 °C.

b 0.8 mmol of K_4_[Fe(CN)_6_]·3H_2_O was used and 1,4-dicyanobenzene was generated.

### Mizoroki–Heck cross-coupling reaction

Inspired by the promising achievements obtained from the cyanation reaction, in the next step, the photocatalytic applicability of the photocatalyst was evaluated in Mizoroki–Heck reaction under visible light irradiation.

To discover the best reaction conditions, various factors comprising the solvent, base, catalyst loading, and light source were screened to optimize the conditions for the benchmark Mizoroki–Heck reaction of iodobenzene and *n*-butyl acrylate under visible light irradiation at room temperature (Table 3, entries 1–23). The results of these experiments illustrated that 0.5 mol% of ZnCo_2_O_4_@g-C_3_N_4_@Cu, K_3_PO_4_, EtOH and 20 W white LED can be selected as the optimal reaction conditions (Table 3, entry 14). The separately conducted control experiments in dark conditions, in the absence of the photocatalyst, and without using base were accompanied with no progress in the reaction, even after 24 h (Table 3, entries 24–26). Thereafter, the photocatalytic activity of Cu(OAc)_2_, g-C_3_N_4_, and ZnCo_2_O_4_@g-C_3_N_4_ were investigated in the model reaction (Table 3, entries 27–29). As observed, trace amount of the product was achieved by using Cu(OAc)_2_ as a photocatalyst (Table 3, entry 27). Also, no product was attained by performing the same model reaction using g-C_3_N_4_ (Table 3, entry 28). In addition, when the reaction was done over ZnCo_2_O_4_@g-C_3_N_4_, the obtained yield was not satisfying and only 20% of the desired product was generated after 24 h (Table 3, entry 29).

**Table 3:** Screening the reaction conditions for the visible light-induced Mizoroki–Heck reaction of iodobenzene with *n*-butyl acrylate over ZnCo_2_O_4_@g-C_3_N_4_@Cu

Entry	Catalyst amount^a^ (mol%)	Base	Solvent	Light source	Time (h)	Isolated yield^b^ (%)
1	0.4	Et_3_N	H_2_O : EtOH (1 : 1)	White LED (20 W)	3	30
2	0.4	Et_3_N	DMF	White LED (20 W)	4	30
3	0.4	Et_3_N	EtOH	White LED (20 W)	5	50
4	0.4	Et_3_N	H_2_O	White LED (20 W)	24	—
5	0.4	Et_3_N	CH_3_CN	White LED (20 W)	3	Trace
6	0.4	Et_3_N	EtOAc	White LED (20 W)	4	30
7	0.4	Et_3_N	HOAc	White LED (20 W)	4	40
8	0.4	Et_3_N	Toluene	White LED (20 W)	24	Trace
9	0.4	NaOH	EtOH	White LED (20 W)	3	20
10	0.4	K_2_CO_3_	EtOH	White LED (20 W)	4	40
11	0.4	Na_2_CO_3_	EtOH	White LED (20 W)	5	Trace
12	0.4	K_3_PO_4_	EtOH	White LED (20 W)	7	70
13	0.45	K_3_PO_4_	EtOH	White LED (20 W)	**6**	85
**14**	**0.5**	**K**_**3**_ **PO**_**4**_	**EtOH**	**White LED (20 W)**	**6**	**92**
15	0.6	K_3_PO_4_	EtOH	White LED (20 W)	**6**	92
16	0.7	K_3_PO_4_	EtOH	White LED (20 W)	9	81
17	0.5	K_3_PO_4_	EtOH	Blue LED (20 W)	8	30
18	0.5	K_3_PO_4_	EtOH	Green LED (20 W)	6	50
19	0.5	K_3_PO_4_	EtOH	Yellow LED (40 W)	8	42
20	0.5	K_3_PO_4_	EtOH	Sunlight^c^	7	60
21	0.5	K_3_PO_4_	EtOH	White LED (10 W)	9	30
22	0.5	K_3_PO_4_	EtOH	White LED (15 W)	6	40
23	0.5	K_3_PO_4_	EtOH	White LED (40 W)	6	92
24	0.5	K_3_PO_4_	EtOH	Dark	24	—
25	0	K_3_PO_4_	EtOH	White LED (20 W)	24	—
26	0.5	—	EtOH	White LED (20 W)	24	—
27^d^	0.5	K_3_PO_4_	EtOH	White LED (20 W)	24	Trace
28^e^	0.5	K_3_PO_4_	EtOH	White LED (20 W)	24	—
29^f^	0.5	K_3_PO_4_	EtOH	White LED (20 W)	24	20

a Based on copper content (except for entries 25, 28 and 29).

b Reaction conditions: iodobenzene (1 mmol), base (2 mmol), *n*-butylacrylate (1.3 mmol), solvent (4 mL), and ZnCo_2_O_4_@g-C_3_N_4_@Cu (except for entries 25 and 27–29), under visible light irradiation. During all experiments, to prevent any photothermal effect, the reaction vessel was immersed in a water bath maintained at 25 °C.

c This experiment was accomplished in Birjand city (Iran), in a summer day, from 9:00 a.m. to 15:00 p.m., at 25 °C.

d Reaction was accomplished by using Cu(OAc)_2_ as the catalyst.

e Reaction was accomplished by using g-C_3_N_4_ as the catalyst.

f Reaction was done using ZnCo_2_O_4_@g-C_3_N_4_ as the catalyst.

To further study the scope and limitations of Mizoroki–Heck reaction over ZnCo_2_O_4_@g-C_3_N_4_@Cu, various substituted aryl halides were chosen and underwent the reaction with various acrylates under the optimal reaction conditions (Table 4). As indicated in Table 4, a variety of aryl halides including aryl iodides (Table 4, entries 1–6), aryl bromides (Table 4, entries 7–10) and aryl chlorides (Table 4, entries 11–13) as the most challenging halide compounds, which are much more economical and attainable than other aryl counterparts, participates in Mizoroki–Heck reaction to deliver the desired products in good to high yields. In all of these experiments, the reaction medium is clean and any side-product was not detected.

**Table 4:** Substrate scope for the Mizoroki–Heck reaction over ZnCo_2_O_4_@g-C_3_N_4_@Cu under visible light illumination

Entry	Aryl halide	Olefin	Time (h)	Isolated yield^a^ (%)
1	PhI	CH_2_=CH–CO_2_Bu^*n*^	6	92
2	PhI	CH_2_=CH–CO_2_Me	7	91
3	PhI	CH_2_=CH–CO_2_Et	7	90
4	PhI	CH_2_=C(Me)–CO_2_Me	5	92
5	4-MeOC_6_H_4_I	CH_2_=CH–CO_2_Bu^*n*^	10	86
6	4-ClC_6_H_4_I	CH_2_=CH–CO_2_Bu^*n*^	9	82
7	PhBr	CH_2_=CH–CO_2_Bu^*n*^	8	90
8	4-O_2_NC_6_H_4_Br	CH_2_=CH–CO_2_Bu^*n*^	7	83
9	4-NCC_6_H_4_Br	CH_2_=CH–CO_2_Bu^*n*^	7	81
10	4-MeOC_6_H_4_Br	CH_2_=CH–CO_2_Bu^*n*^	12	84
11	PhCl	CH_2_=CH–CO_2_Bu^*n*^	12	76
12	4-O_2_NC_6_H_4_Cl	CH_2_=CH–CO_2_Bu^*n*^	11	75
13	4-NCC_6_H_4_Cl	CH_2_=CH–CO_2_Bu^*n*^	11	71

a Reaction conditions: aryl halide (1 mmol), olefin (1.3 mmol), K_3_PO_4_ (2 mmol), ZnCo_2_O_4_@g-C_3_N_4_@Cu (0.5 mol%) and EtOH (4 mL), using white LED lamp (20 W). To prevent any photothermal effect, the reaction vessel was immersed in a water bath maintained at 25 °C.

### Studies of the heterogeneity of ZnCo_2_O_4_@g-C_3_N_4_@Cu

**Fig. 5:**
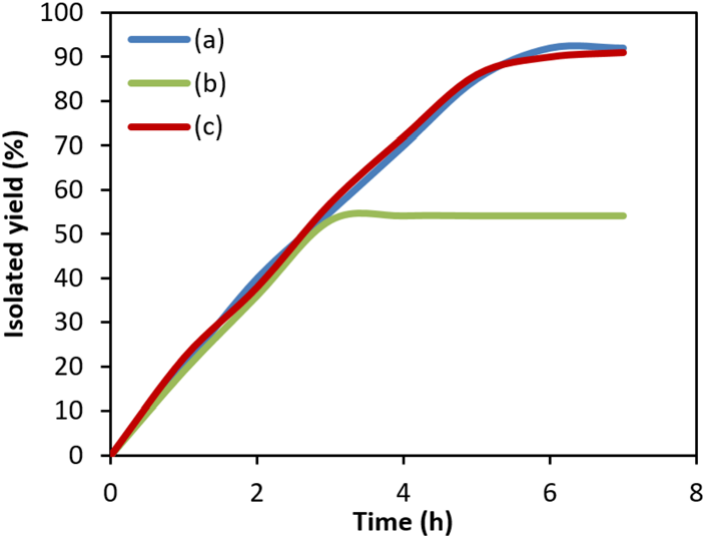
Reaction progress *vs.* the irradiation time for Mizoroki–Heck model reaction in (a) normal conditions, (b) filtration experiment and (c) poisoning test.

To ascertain whether ZnCo_2_O_4_@g-C_3_N_4_@Cu acts in a real heterogeneous pathway or not, poisoning and filtration tests were performed. For filtration experiment, the model Mizoroki–Heck reaction was done under the optimum conditions and once half of the reaction time has passed, the photocatalyst was isolated from the reaction solution and the reaction was permitted to proceed with no catalyst. After continuing the reaction for further 10 hours, no more product was generated, which clearly confirmed that any homogeneous catalyst is not present in the reaction medium (Fig. 5b). ICP-OES analysis of the filtrate was associated with a negligible content of Cu (<0.1% of the total Cu amount). Poisoning test was done by conducting the model reaction of Mizoroki–Heck in the presence of S_8_ (0.07 g) as a metal scavenger. As it is apparent, no significant change in the reaction rate was witnessed in the presence of the scavenger (Fig. 5c). These results showed that ZnCo_2_O_4_@g-C_3_N_4_@Cu has a truly heterogeneous function in this process.

### Investigation of a large-scale photocatalytic process

The resulting products in the present work are extremely applicable as the basic building blocks in chemical manufacturing industries. Consequently, to evaluate the practical synthetic applications of this method, the model cyanation and Mizoroki–Heck reactions were separately investigated in a scaled-up procedure (50 times), under optimal conditions. Interestingly, the cyanation reaction proceeded in 8 h, affording 91% yield of the product, while the Mizoroki–Heck reaction was associated with 90% of the desired product after 6 h.

### Studies of the stability and reusability of the photocatalyst in cyanation and Mizoroki–Heck cross-coupling reactions

**Fig. 6:**
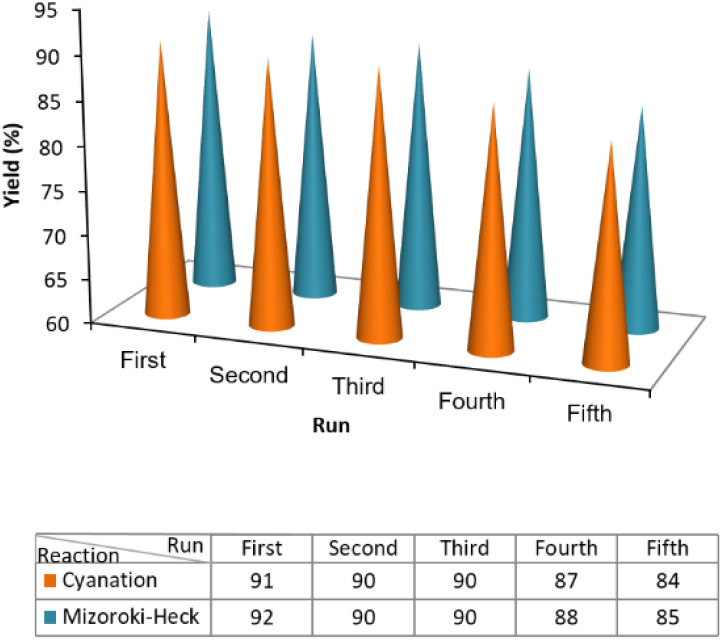
Recycling of the photocatalyst in the model cyanation and Mizoroki–Heck reactions under optimal conditions. The reactions were monitored 7 and 6 h for the cyanation and Mizoroki–Heck reactions, respectively.

In heterogeneous photocatalysis, recovering and recycling are very important aspects, particularly for environmental and practical purposes. In this line, the recyclability of the photocatalyst was evaluated for the cyanation and Mizoroki–Heck model reactions under the optimum conditions. As shown in Fig. 6, in both cyanation and Mizoroki–Heck reactions, ZnCo_2_O_4_@g-C_3_N_4_@Cu could be recovered and reused for at least five consecutive runs and the final products were obtained with respectively 84% and 85% yields after the 5th cycle. These results verified the appreciable durability of the photocatalyst.

**Fig. 7:**
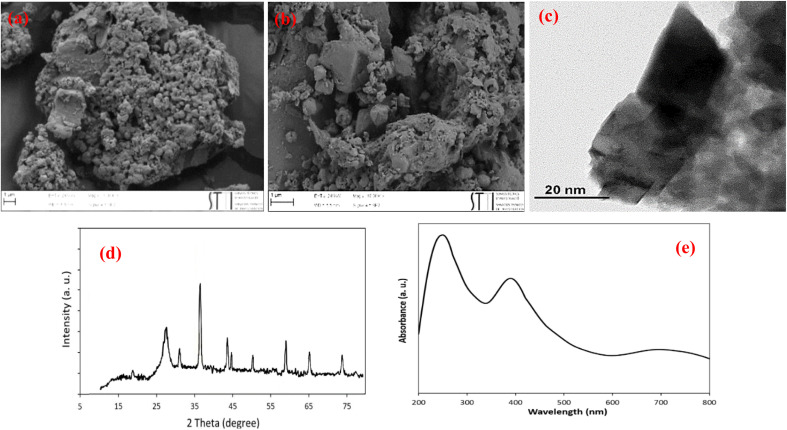
(a, b) FESEM, (c) TEM, (d) XRD pattern and (e) UV-vis DRS of ZnCo_2_O_4_@g-C_3_N_4_@Cu after five times reuses.

According to the FESEM and TEM images (Fig. 7a–c), XRD analysis (Fig. 7d) and UV-vis DRS results (Fig. 7e), the chemical, morphological and optical structure of ZnCo_2_O_4_@g-C_3_N_4_@Cu remained largely intact after five consecutive reuses.

ICP-OES analysis of the reused catalyst 5th cycles showed that the recovered catalyst contains 0.50 mmol of Cu per 1 g of the catalyst. This means that the amount of copper leached from the surface of the catalyst is negligible.

These results confirmed the outstanding stability and durability of the photocatalyst for visible-light driven cyanation and Mizoroki–Heck cross-coupling reactions.

### Comparative study

The advantages of the presented photocatalyst were compared with previously studied visible light-induced photocatalytic systems in similar coupling transformations (Table 5). While each of these approaches has their own merits, they often suffer from one or more of the following shortcomings including the strict reaction conditions, use of hazardous solvents, noxious/expensive/inaccessible light source, large amounts of costly Pd-based photocatalysts, higher reaction temperatures, longer reaction times and lower product yields. These results revealed the superlative photocatalytic activity of ZnCo_2_O_4_@g-C_3_N_4_@Cu, arising from the synergistic optical properties of ZnCo_2_O_4_, g-C_3_N_4_, and Cu. The current approach effectively promoted the cyanation and Mizoroki–Heck reactions through an eco-friendly process, and is highly effective for a broad range of corresponding derivatives.

**Table 5:** Comparison of the photocatalytic activity of ZnCo_2_O_4_@g-C_3_N_4_@Cu with the previously reported photocatalyzed cyanation and Mizoroki–Heck cross-coupling reactions under visible light

Entry^ref^	Reaction	Catalyst (amount)	Reaction condition	Time (h)	Yield^a^ (%)
1 (ref. 81)	Cyanation	Pd@CeO_2_ (100 mg/3 wt%)	NaOAc, DMF/i-PrOH = 7 : 1, air atmosphere, 55 °C, 500 W halogen tungsten lamp (400–750 nm)	12	58.6
2^This work^		ZnCo_2_O_4_@g-C_3_N_4_@Cu (0.7 mol%)	Et_3_N, H_2_O : EtOH (1 : 1), white LED lamp irradiation (20 W), 25 °C	6	92
3 (ref. 59)	Mizoroki–Heck	Pd-rGO/CNT/CaFe_2_O_4_ (0.1 mol%)	TEA, DMA, 25 °C, visible light (250 W mercury lamp with a UV cut-off filter)	5	67
4 (ref. 60)		Pd/CNCs^b^ (50 mg)	K_2_CO_3_, DMF, 40 °C, 300 W Xe lamp (400–800 nm), inert atmosphere	3	99
5 (ref. 61)		AuPd@NRCN^c^ (15 mg)	Et_3_N, DMF, r.t., visible light irradiation- blue LED lamp (intensity: 0.75 W cm^−2^), air atmosphere	34	60
6 (ref. 62)		Pd/SiC	K_2_CO_3_, DMF, 40 °C, xenon lamp (300 W), Ar atmosphere	4	99.6
7 (ref. 63)		HP-T@Au-Fe_3_O_4_^d^	K_2_CO_3_, neat, 40 °C, visible light, air atmosphere	2	91
8 (ref. 64)		NiCu@CNOs^e^ (20 mg)	K_2_CO_3_, H_2_O, r.t., mercury-vapor lamp (400 W), air atmosphere	0.83	97
9 (ref. 65)		Pd(OAc)_2_ (2.2 mg)	Et_3_N, DMF, r.t., blue LED	24	99
		Ru(bpy)_3_Cl_2_·6H_2_O (7.5 mg)			
10^This work^		ZnCo_2_O_4_@g-C_3_N_4_@Cu (0.5 mol%)	K_3_PO_4_, EtOH, 25 °C, white LED lamp irradiation (20 W), air atmosphere	7	91

a The model cyanation reaction is iodobenzene and K_4_[Fe(CN)_6_]·3H_2_O (entries 1 and 2). The model Mizoroki–Heck reaction is iodobenzene and methyl acrylate (entries 2–8 and 10) or ethyl acrylate (entry 9).

b CNCs: carbon nanocoils.

c NRCN: N-rich carbon nitride.

d HP-T: hexaphenylbenzene-thiophene.

e CNOs: carbon nano-onions.

### Photocatalytic mechanism

**Fig. 8:**
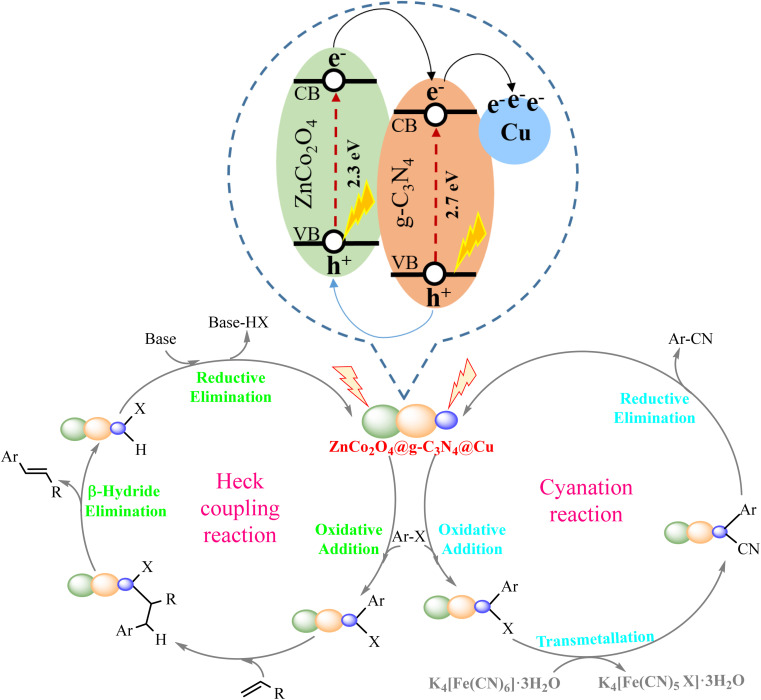
A proposed photocatalytic mechanism for the visible light-induced cyanation and Mizoroki–Heck cross-coupling reactions over ZnCo_2_O_4_@g-C_3_N_4_@Cu.

To insights into a proper reaction mechanism, control experiment was conducted by monitoring the model cyanation and Mizoroki–Heck cross-coupling reactions in the presence of 2,2,6,6-tetramethylpiperidine-1-oxyl (TEMPO) as a radical scavenger. The results showed that after involving the radical trapping agent (aryl halide/TEMPO; 1 : 2) the visible light-driven reactions were completely quenched and no product was obtained. Likewise, performing the model coupling reactions in the absence of visible light source was accompanied with no desired product (Table 1, entry 24 and Table 3, entry 24). These results confirmed that such reactions are likely to involve a radical process and light is required to complete the reactions. Having these results in hand and based on the literature review on the heterogeneous photocatalytic cyanation^81,82^ and Mizoroki–Heck^59–63^ cross-coupling reactions, a proposed reaction mechanism was presented for the visible light-induced cyanation and Mizoroki–Heck cross-coupling reactions (Fig. 8). Upon visible light illumination, both ZnCo_2_O_4_ and g-C_3_N_4_ were aroused simultaneously and the electrons were excited from the valence band (VB) and transferred to the conduction band (CB) on both ZnCo_2_O_4_ and g-C_3_N_4_ to produce the photo generated electrons and holes. The photogenerated electrons accumulated on the CB of ZnCo_2_O_4_ can easily transfer to the CB of g-C_3_N_4_ (due to the low potential energy) with the assistance of the internal electric field owing to the formation of a p–n heterojunction between ZnCo_2_O_4_ and g-C_3_N_4_.^83^ The existing electrons in the CB of g-C_3_N_4_ were simultaneously injected to Cu. These NPs are an effective material for trapping the photogenerated electrons because of their electron reservoir capacity.^84^ Meanwhile, photogenerated holes in the VB of g-C_3_N_4_ can easily immigrate into the VB of ZnCo_2_O_4_. The appropriate transformations of the charge carriers along the p–n heterojunction interfaces of the photocatalyst resulted in the efficient separation of photogenerated electron and hole pairs, which could suitably extend the lifetime of the corresponding excited electrons and holes. Energetic electrons concentrated on the surface of Cu NPs, caused it to undergo oxidative addition with aryl halides. This phenomenon facilitated the cleavage of C–X bonds in aryl halide and formed Ar–Cu–X complex. In Mizoroki–Heck cross-coupling reaction, this step was followed by fast insertion of alkene to Ar–Cu–X complex. The final product could be achieved by β-hydrogen elimination, and the catalyst could be regenerated under basic conditions.^59–63^ In the case of cyanation cross-coupling reaction, after the formation of Ar–Cu–X complex trough an oxidative addition process, cyanide anion of K_4_[Fe(CN)_6_]·3H_2_O underwent transmetallation reaction to provide a transient organometallic complex intermediate. Finally, aryl nitrile was produced from the complex by reductive elimination and the re-generated catalyst re-entered the catalytic cycle.^81^

## Experimental

### Fabrication of the photocatalyst

#### Synthesis of g-C_3_N_4_ nanosheets

The g-C_3_N_4_ nanosheets were prepared by slightly modifying the previously reported consecutive polymerization and liquid exfoliation methods.^85^ Initially, the calcination process of melamine (5 g) was done at 550 °C (5 °C per min) for about 3 h in air. The resultant yellow solid was allowed to reach room temperature. Then, it was completely milled to turn from agglomerated state into a uniform powder. Subsequently, 0.1 g of as-prepared g-C_3_N_4_ bulk powder was mixed with deionized water (100 mL) and subjected to ultrasonic treatment for 6 h. Then, the suspension was centrifuged at 3000 rpm for the elimination of the un-exfoliated g-C_3_N_4_ and the obtained g-C_3_N_4_ nanosheets were collected and dried.

#### Synthesis of ZnCo_2_O_4_/g-C_3_N_4_

Initially, 0.28 mmol of Co(NO_3_)_2_·6H_2_O and 0.14 mmol of Zn(NO_3_)_2_·6H_2_O were dispersed in distilled water (75 mL) for 20 min. Afterwards, a solution of NaOH (2 M) was added dropwise (1 mL per min) into the suspension. The addition of NaOH solution was stopped by the adjustment of the pH solution at 10. Then, the solution was stirred intensively at room temperature for 25 min. In the next step, the obtained suspension was charged with 0.5 g of g-C_3_N_4_ nanosheets and refluxed for 1 h. The resultant mixture was centrifuged, washed temporarily with deionized water and dried in a vacuum oven. Eventually, the obtained sample was heated for 2 h in a furnace at 350 °C (5 °C per min) to yield ZnCo_2_O_4_@g-C_3_N_4._

#### Synthesis of ZnCo_2_O_4_@g-C_3_N_4_@Cu

A suspension containing 1 g of pre-prepared ZnCo_2_O_4_@g-C_3_N_4_ in EtOH (30 mL) was sonicated for 30 min. Following this, a solution of Cu(OAc)_2_ (2 mmol) in 15 mL EtOH was added drop by drop to the mixture, while constant stirring was applied for 1 h. Subsequently, an aqueous solution of NaBH_4_ (30 mL, 0.1 M) was incrementally added to the mixture and stirred vigorously for 3.5 h. The resultant ZnCo_2_O_4_@g-C_3_N_4_@Cu was separated through centrifugation at 1000 rpm, and washed with distilled water (2 × 15 mL) and EtOH (3 × 15 mL), before drying in a vacuum at 60 °C.

#### General procedure for photocatalytic cyanation reaction using ZnCo_2_O_4_@g-C_3_N_4_@Cu under visible light

ZnCo_2_O_4_@g-C_3_N_4_@Cu (0.7 mol%) was added to a 10 mL Pyrex test tube charged with a mixture of EtOH : H_2_O (1 : 1, 4 mL), aryl halide (1 mmol), K_4_[Fe(CN)_6_]·3H_2_O (0.4 mmol), and Et_3_N (1 mmol). In all experiments, to prevent any photothermal heating effect, the reaction vial was immersed in a water bath maintained at 25 °C. Thereafter, the reaction container was exposed to a white LED lamp (20 W) at a distance of 10 cm. After stirring for an appropriate time mentioned in Table 2, the reaction mixture was diluted with EtOH (5 mL), and the catalyst was separated by centrifugation (1000 rpm), washed with EtOH (2 × 5 mL) and air-dried to prepare for the subsequent reaction process. The solvent of combined organic layer was removed under the rotary evaporation to afford the crude product. The desired pure product was then obtained using a silica gel column chromatography technique (*n*-hexane : ethyl acetate; 6 : 1).

#### General procedure for photocatalytic Mizoroki–Heck reaction using ZnCo_2_O_4_@g-C_3_N_4_@Cu under visible light irradiation

ZnCo_2_O_4_@g-C_3_N_4_@Cu (0.5 mol%) was added to a 10 mL Pyrex test tube containing a mixture of olefin (1.3 mmol), aryl halide (1 mmol), K_3_PO_4_ (2 mmol) and EtOH (4 mL). In all experiments, to prevent any photothermal heating effect, the reaction vial was immersed in a water bath maintained at 25 °C. Thereafter, the reaction container was exposed to a white LED lamp (20 W) at a distance of 10 cm. After stirring for an appropriate time mentioned in Table 4, the reaction mixture was diluted with EtOH (5 mL), and the catalyst was isolated by centrifugation (1000 rpm), washed with EtOH (2 × 5 mL) and air-dried to use in the subsequent reaction. Then, the residuals of solvent were removed by vacuum evaporation. The pure product was then afforded using a silica gel column chromatography technique (*n*-hexane : ethyl acetate; 50 : 1).

## Conclusions

In this study, ZnCo_2_O_4_@g-C_3_N_4_@Cu was found as a superb photocatalyst to promote the visible light-driven cyanation and Mizoroki–Heck reactions of a wide range of aryl halides including aryl iodides, aryl bromides, and aryl chlorides (as the challenging class of cross-coupling reactions with few precedents), with K_4_[Fe(CN)_6_]·3H_2_O and olefins, respectively, at room temperature. The synergistic optical effect among ZnCo_2_O_4_, g-C_3_N_4_, and Cu is responsible for the enhanced photocatalytic performance of the photocatalyst. The poisoning and filtration tests were accomplished to verify the actual heterogeneity and durability of ZnCo_2_O_4_@g-C_3_N_4_@Cu under the reaction conditions. The recovered photocatalyst can be readily recycled for at least five runs while maintaining its catalytic activity and morphology. Applying an economical and benign light source, facile and low-cost photocatalytic approach, no requirement for heat or any additives, scalability of the protocol, aerial conditions, and utilizing an eco-benign solvent are the other important merits of this procedure. It is important to note that the current study represents the first report of employing a heterogeneous copper-based photocatalyst for the cyanation reactions of various aryl halides with K_4_[Fe(CN)_6_]·3H_2_O under visible-light irradiation.

## Data availability

The data supporting this article have been included as part of the ESI.†

## Author contributions

Roya Jahanshahi: conceptualization, methodology, investigation, writing-original draft, review and editing. Hadis Hosseini Moghadam: methodology, data curation. Sara Sobhani: supervision, validation, review and editing. Jose' Miguel Sansano: performing the XPS, FESEM and TEM analysis, review and editing.

## Conflicts of interest

There are no conflicts to declare.

## Supplementary Material

RA-014-D4RA04827J-s001
