# Supplementary material for: ZnCo2O4@g-C3N4@Cu as a new and highly efficient heterogeneous photocatalyst for visible light-induced cyanation and Mizoroki–Heck cross-coupling reactions
Source: RSC Adv. 2024 Aug 22;14(36):26424–36. doi: 10.1039/d4ra04827j (PMC11339774; doi:10.1039/d4ra04827j)
Supplement: RA-014-D4RA04827J-s001 [file RA-014-D4RA04827J-s001.pdf]

Supplementary Information (SI) for RSC Advances.
This journal is © The Royal Society of Chemistry 2024

Supporting Information

$ZnCo_2O_4$@g-C_3N_4@Cu as a new and highly efficient heterogeneous photocatalyst for visible light-induced cyanation and Mizoroki-Heck cross-coupling reactions

Roya Jahanshahi,[b] Hadis Hosseini Moghadam,[b] Sara Sobhani*[a,b] and Jose´ Miguel Sansano[c]

Author addresses:

[a] *Department of Chemistry, College of Sciences, Shiraz University, Shiraz 71454, Iran.*

[b]*Department of Chemistry, College of Sciences, University of Birjand, Birjand, Iran.*

**Email address of the corresponding author: ssobhani@birjand.ac.ir*

[c]*Departamento de Química Orgánica, Facultad de Ciencias, Centro de Innovación en Química Avanzada (ORFEO-CINQA) and Instituto de Síntesis Orgánica (ISO), Universidad de Alicante, Apdo. 99, 03080-Alicante, Spain.*

Experimental

Materials and instruments

All chemicals and solvents were purchased from Merck chemical company and were used directly without further treatment. The purity of the products and the progress of the reactions were monitored using TLC on silica-gel Polygram SILG/UV254 plates. FT-IR spectra were recorded with a JASCO FT-IR 460 plus spectrophotometer within the 400-4000 cm^{-1} range using KBr disc at room temperature. X-ray photoelectron spectroscopy (XPS) analyses were carried out using a VG-Microtech Multilab 3000 spectrometer, equipped with an Al anode. The deconvolution of the spectra was carried out by using Gaussian Lorentzian curves. X-ray powder diffraction (XRD) was carried out on an Xpert Pro Panalytical diffractometer (PW1730, PHILIPS company) with Cu Kα radiation (λ = 1.540 Å). Energy-dispersive X-ray spectroscopy (EDS) and elemental mapping were performed using a TESCAN MIRA3 instrument. Transmission electron microscopy (TEM) was done using the TEM microscope JEOL JEM-1400 Plus. FESEM microscopy is performed in a Hitachi model S3000N. The content of copper in the catalyst was determined by OPTIMA 7300DV ICP-OES analyzer. UV-vis diffuse reflectance spectroscopy (DRS) was conducted using a Shimadzu spectrophotometer (UV-2550 model). The NMR spectra were provided by Brucker Avance 300 and 400 MHz instruments in $CDCl_3$ and DMSO-d_6, in the presence of tetramethylsilane as the internal standard and the coupling constants (*J* values) are given in Hz.

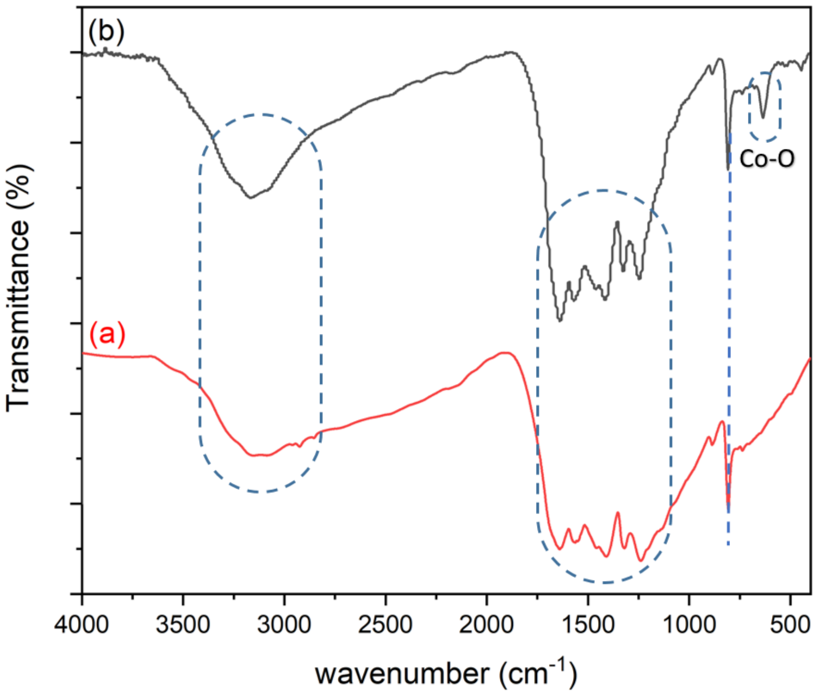

Figure S1. *FT-IR spectra of (a) g-C_3N_4 and (b) $ZnCo_2O_4$@g-C_3N_4@Cu.*

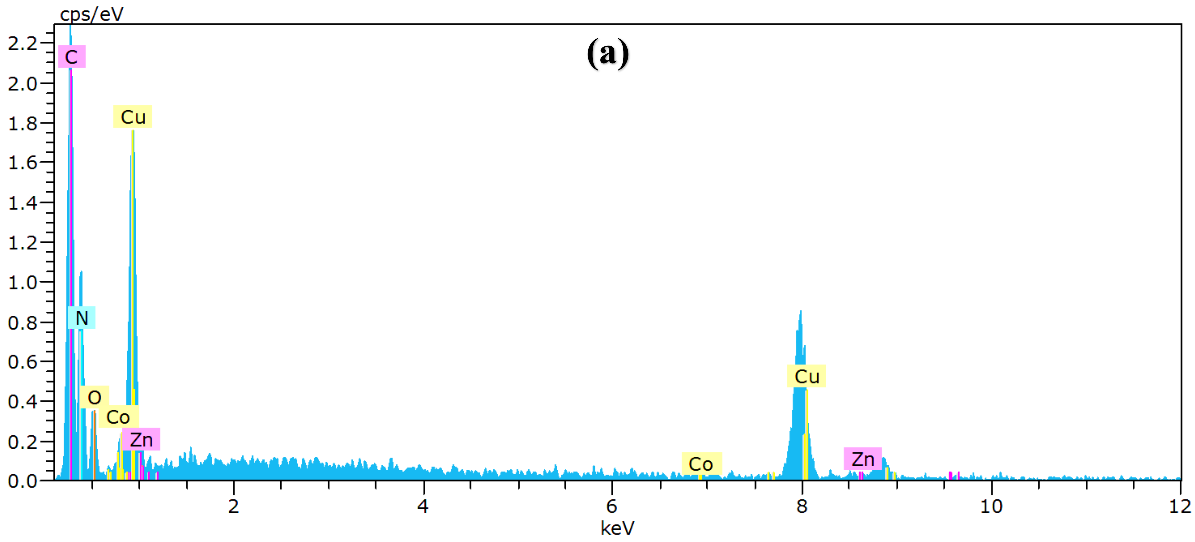
cps/eV
(a)
C
Cu
N
O
Co
Zn
Cu
Co
Zn
keV

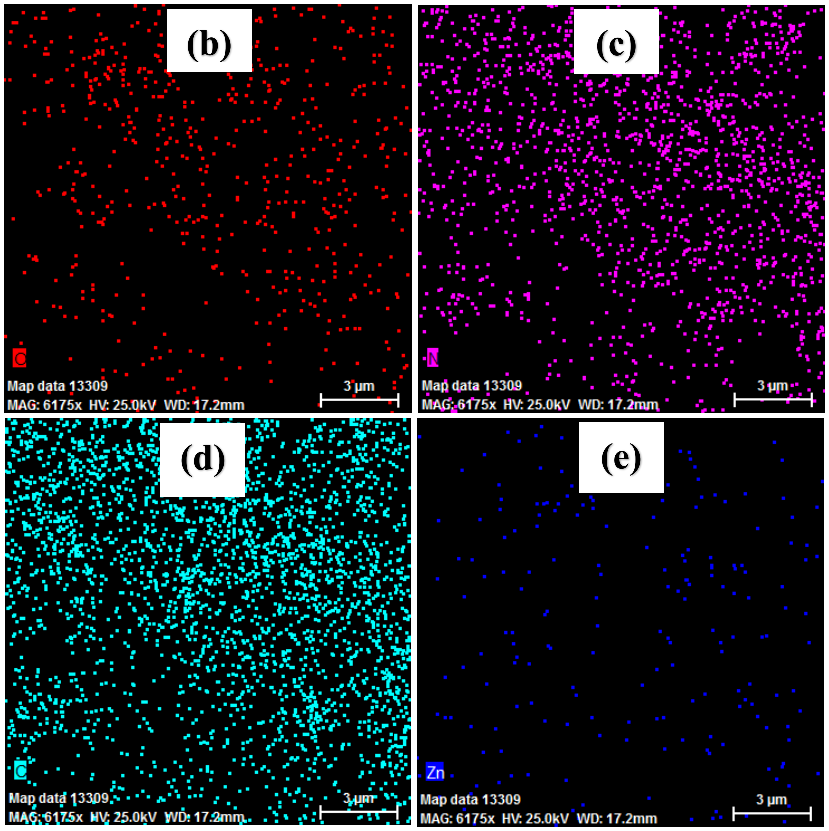
(b)
C
Map data 13309
MAG: 6175x HV: 25.0kV WD: 17.2mm
3 µm
(c)
N
Map data 13309
MAG: 6175x HV: 25.0kV WD: 17.2mm
3 µm
(d)
Map data 13309
MAG: 6175x HV: 25.0kV WD: 17.2mm
3 µm
(e)
Zn
Map data 13309
MAG: 6175x HV: 25.0kV WD: 17.2mm
3 µm

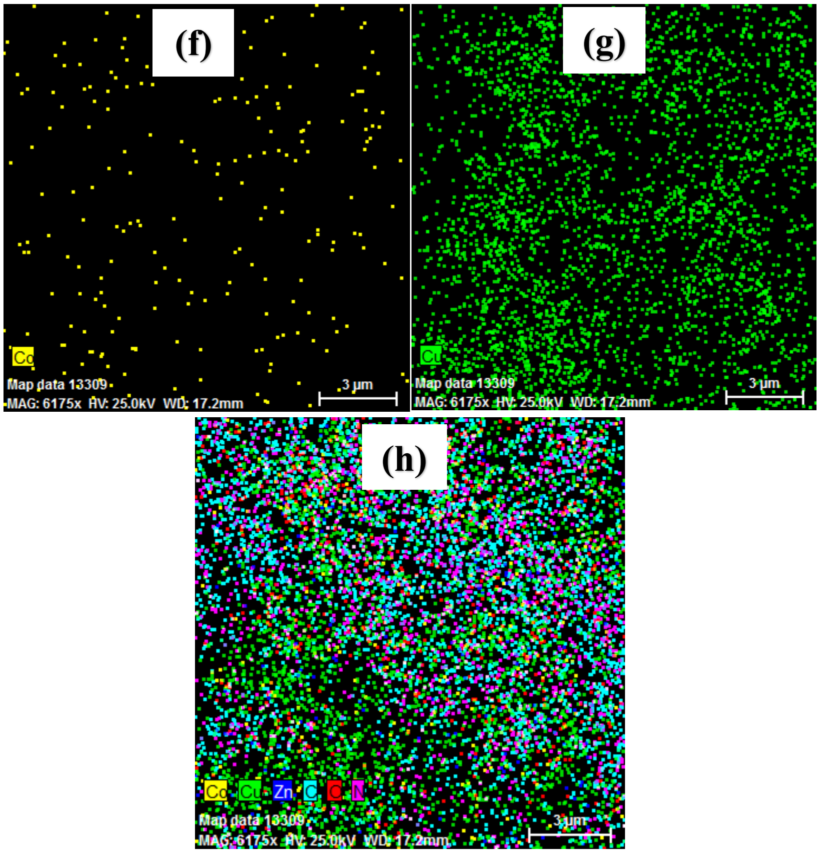

Figure S2. *(a) EDS analysis and elemental mapping images of (b) oxygen (red), (c) nitrogen (purple), (d) carbon (cyan), (e) zinc (blue), (f) cobalt (yellow), (g) copper (green) and (h) the overlapping of O, N, C, Zn, Co and Cu elements in* $ZnCo_2O_4$@g-C_3N_4@Cu.

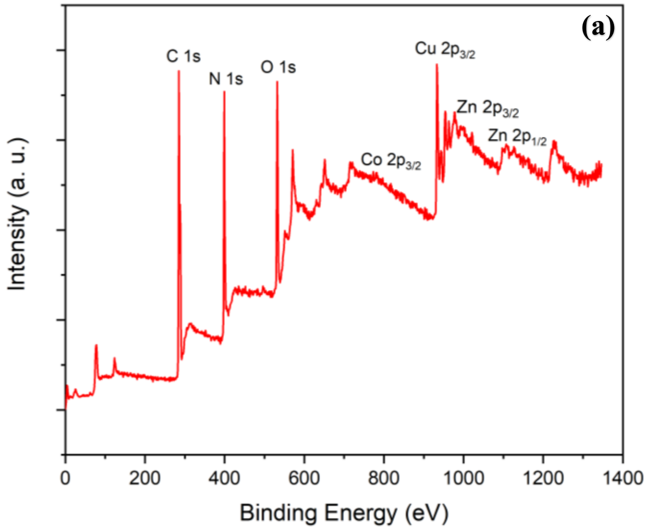
(a)
C 1s
N 1s
O 1s
Cu 2p3/2
Zn 2p3/2
Zn 2p1/2
Co 2p3/2
Intensity (a. u.)
0
200
400
600
800
1000
1200
1400
Binding Energy (eV)

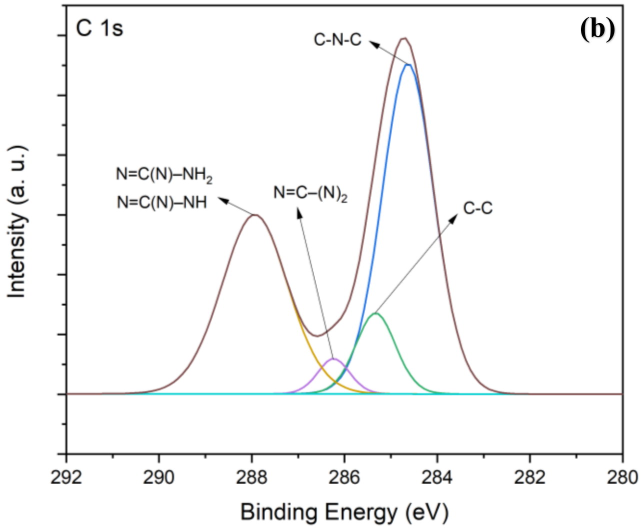
C 1s
(b)
C-N-C
N=C(N)–NH2
N=C(N)–NH
N=C–(N)2
C-C
Intensity (a. u.)
292
290
288
286
284
282
280
Binding Energy (eV)

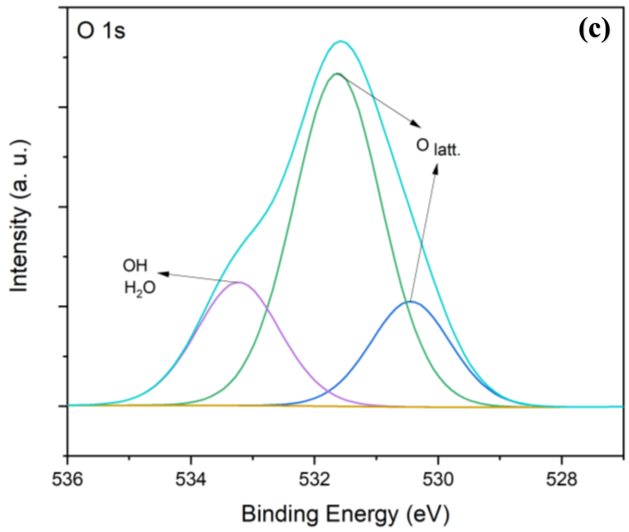
O 1s
(c)
O latt.
OH
H_2O
Intensity (a. u.)
536
534
532
530
528
Binding Energy (eV)

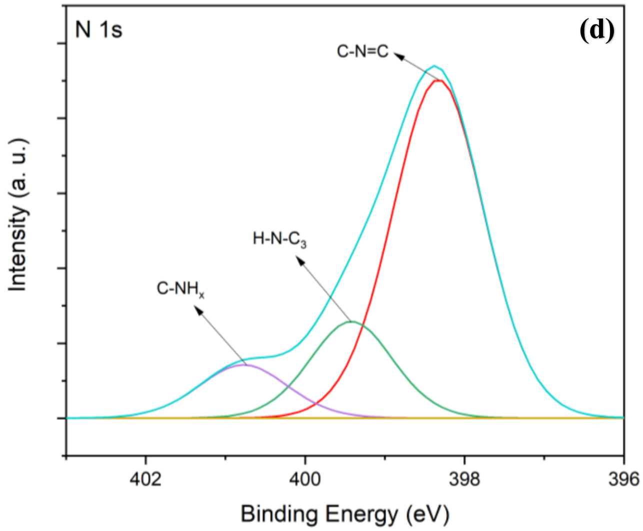
N 1s
(d)
C-N=C
H-N-C_3
C-NH_x
Intensity (a. u.)
402
400
398
396
Binding Energy (eV)

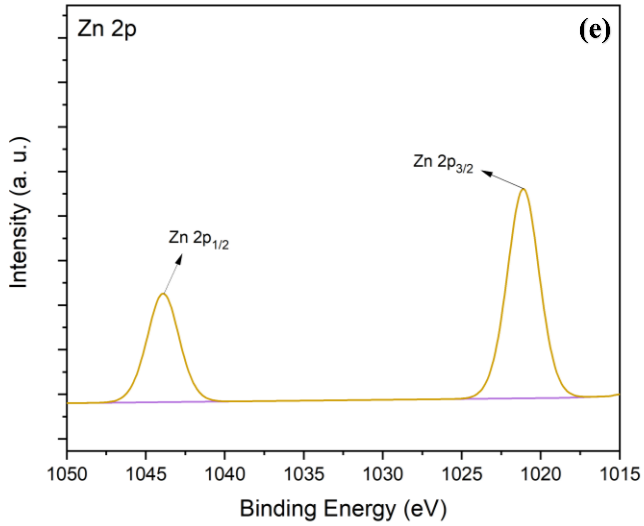
Zn 2p
(e)
Zn $2p_{3/2}$
Zn $2p_{1/2}$
Intensity (a. u.)
1050
1045
1040
1035
1030
1025
1020
1015
Binding Energy (eV)

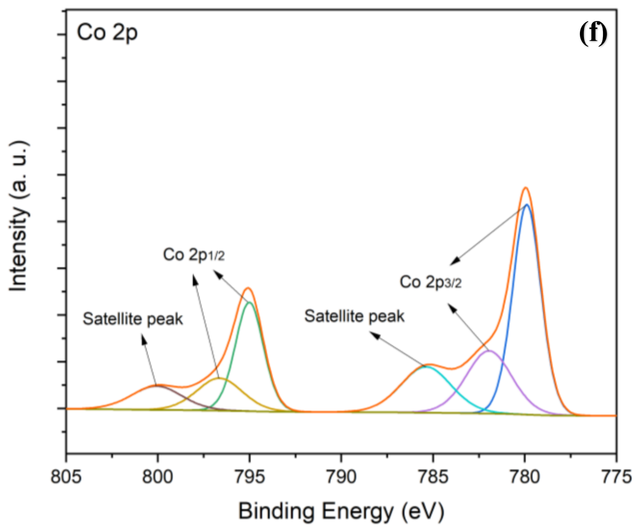

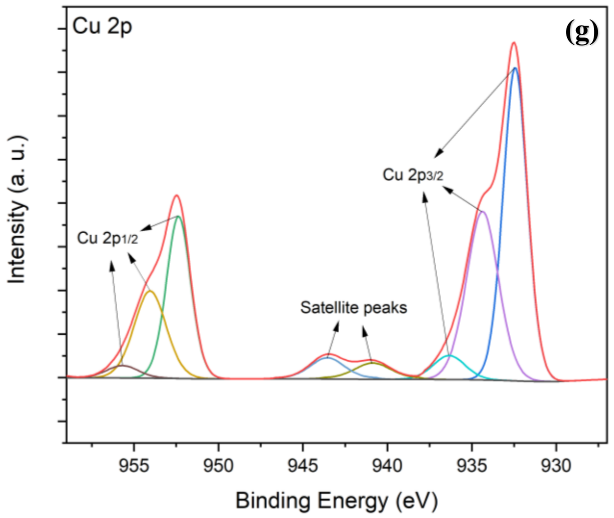

Figure S3. *(a) XPS analysis of $ZnCo_2O_4$@g-C_3N_4@Cu, (b) C 1s, (c) O 1s, (d) N 1s, (e) Zn 2p, (f) Co 2p and (g) Cu 2p.*

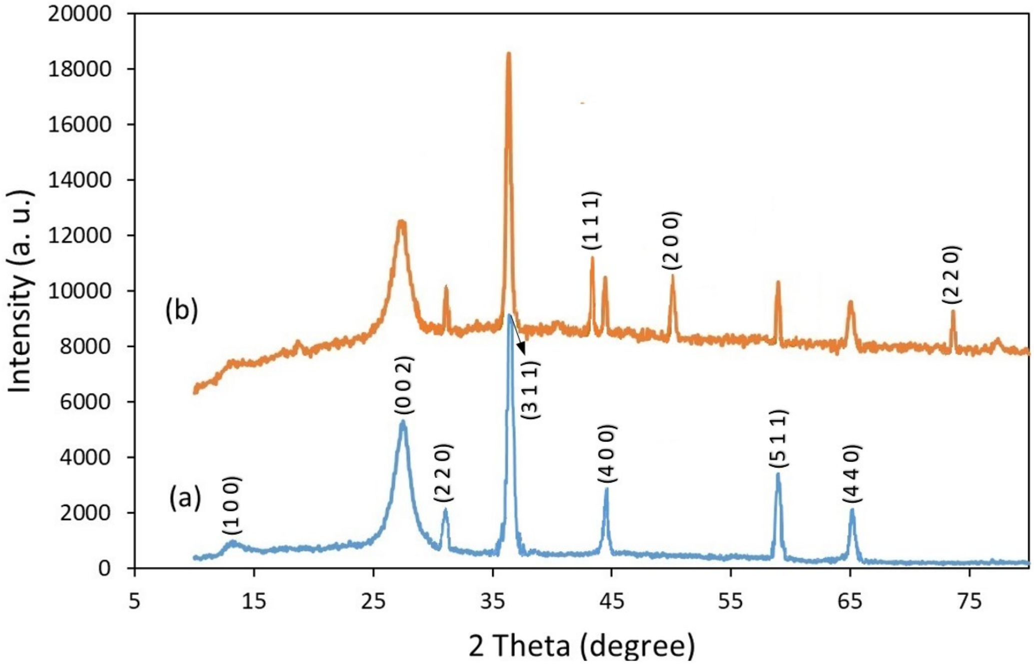

Figure S4. *XRD patterns of (a) $ZnCo_2O_4$@g-C_3N_4 and (b) $ZnCo_2O_4$@g-C_3N_4@Cu.*

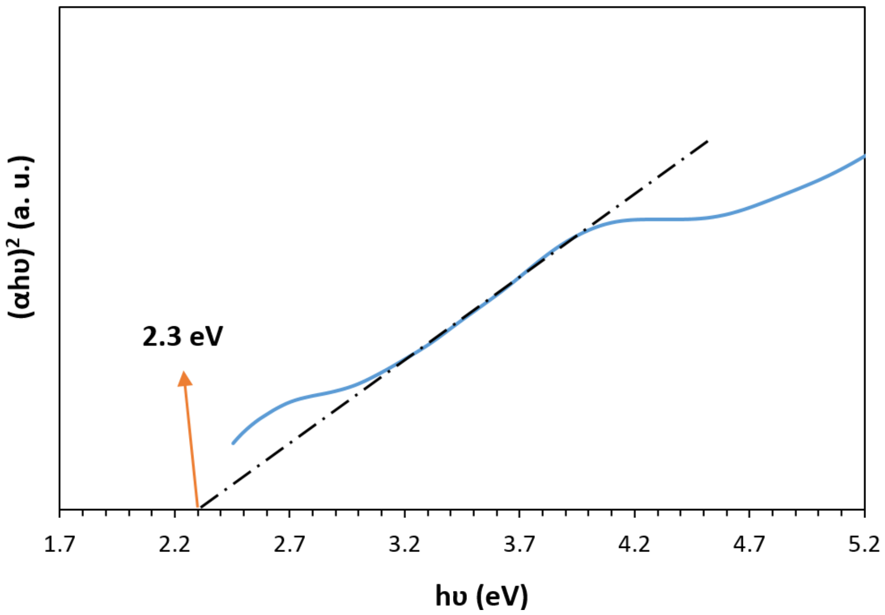

Figure S5. *Tauc plot of $ZnCo_2O_4$@g-C_3N_4@Cu for the estimation of band gap energy.*

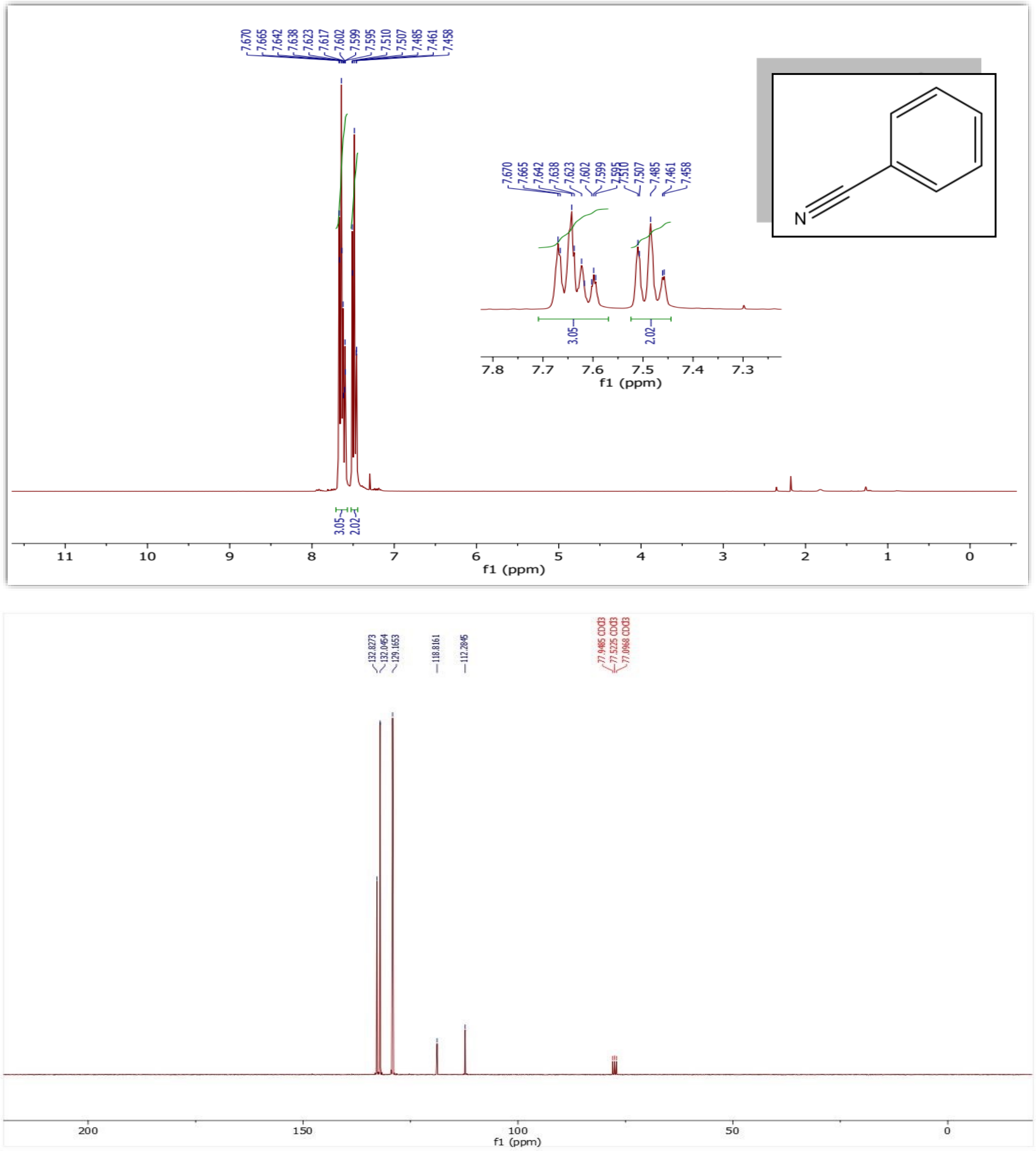

^{1}H NMR and ^{13}C NMR spectra of benzonitrile

^{1}H NMR (300 MHz, $CDCl_3$): δ 7.59-7.67 (m, 3 H), 7.48 (t, *J* = 7.8 Hz, 2 H) ppm; ^{13}C NMR (75 MHz, $CDCl_3$): δ 132.8, 132.0, 129.1, 118.8, 112.2 ppm.

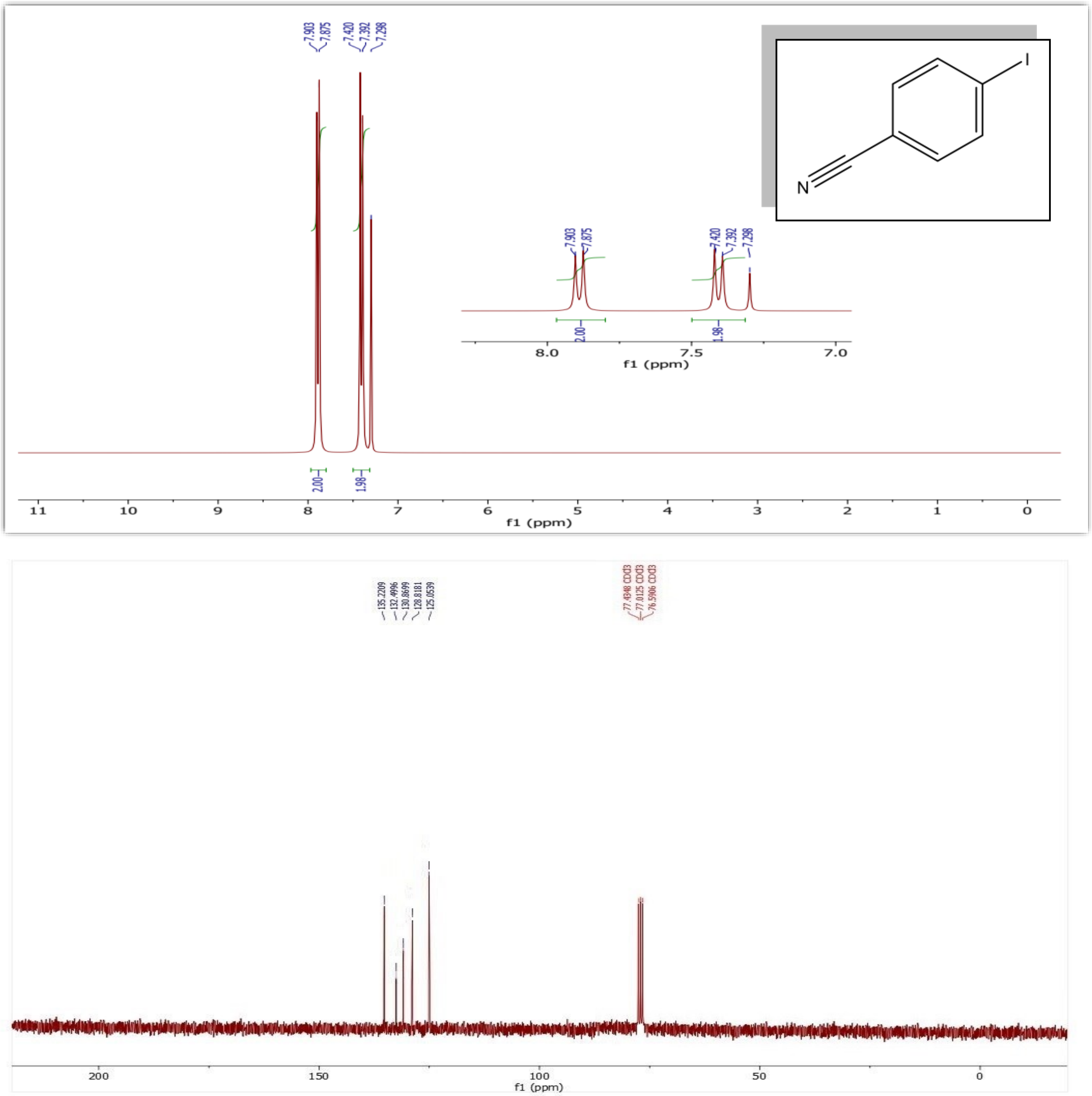

^{1}H NMR and ^{13}C NMR spectra of 4-iodobenzonitrile

^{1}H NMR (300 MHz, $CDCl_3$): *δ* 7.89 (d, *J* = 8.4 Hz, 2 H), 7.40 (d, *J* = 8.4 Hz, 2 H) ppm; ^{13}C NMR (75 MHz, $CDCl_3$): δ 135.2, 132.4, 130.8, 128.8, 125.0 ppm.

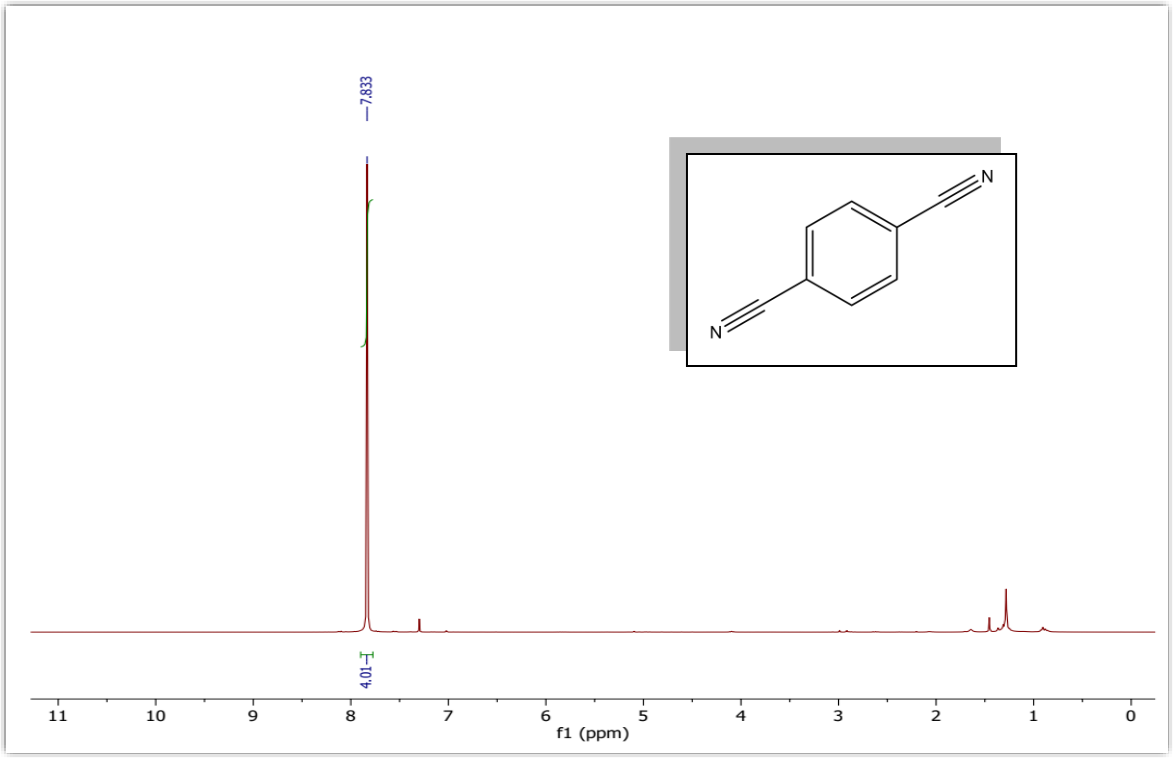

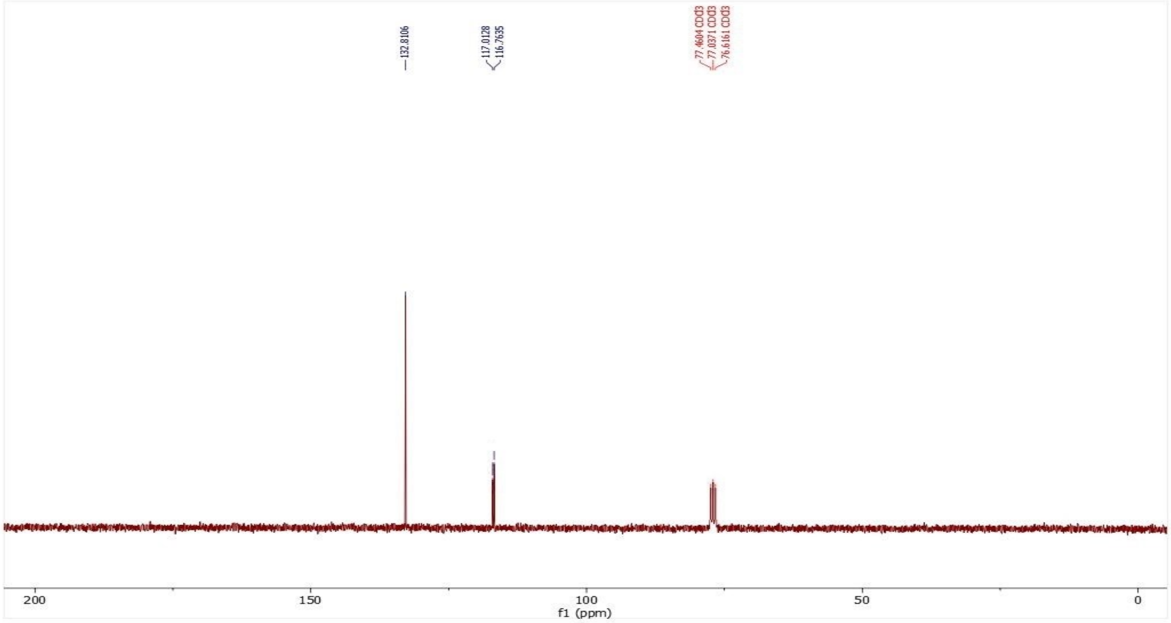

^{1}H NMR and ^{13}C NMR spectra of terephthalonitrile

^{1}H NMR (300 MHz, $CDCl_3$): *δ* 7.83 (s, 4 H) ppm; ^{13}C NMR (75 MHz, $CDCl_3$): δ 132.8, 117.0, 116.7 ppm.

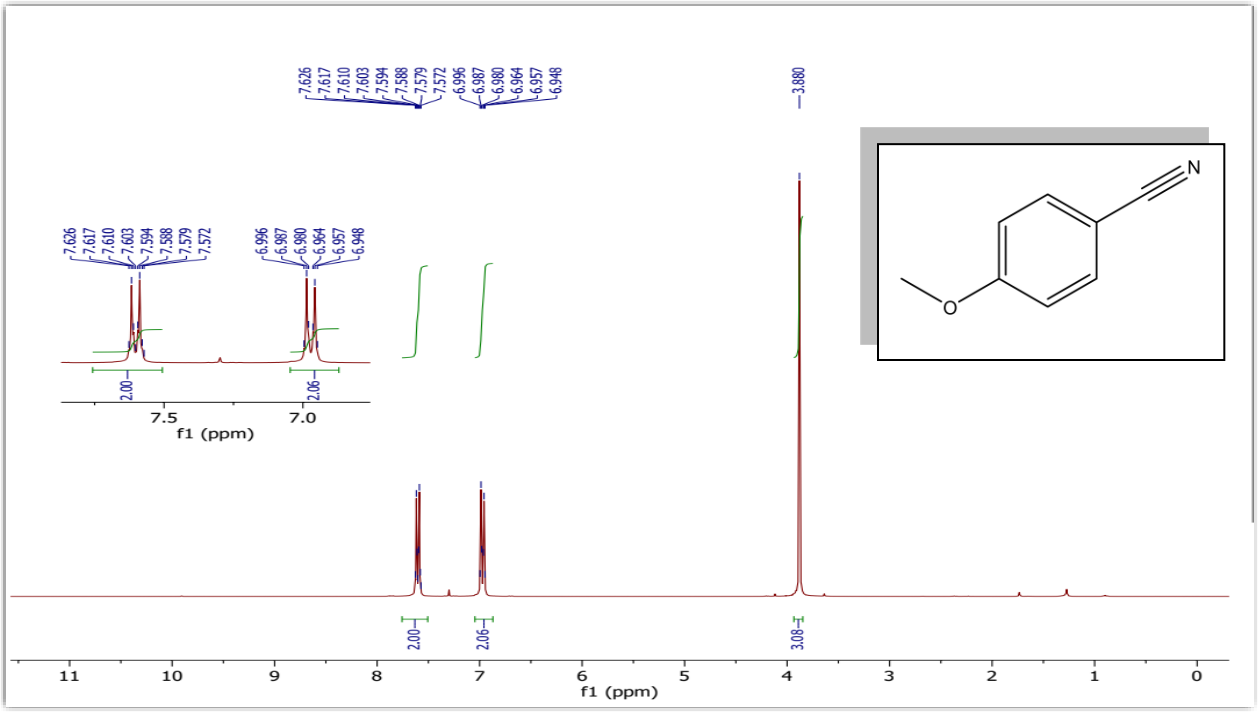

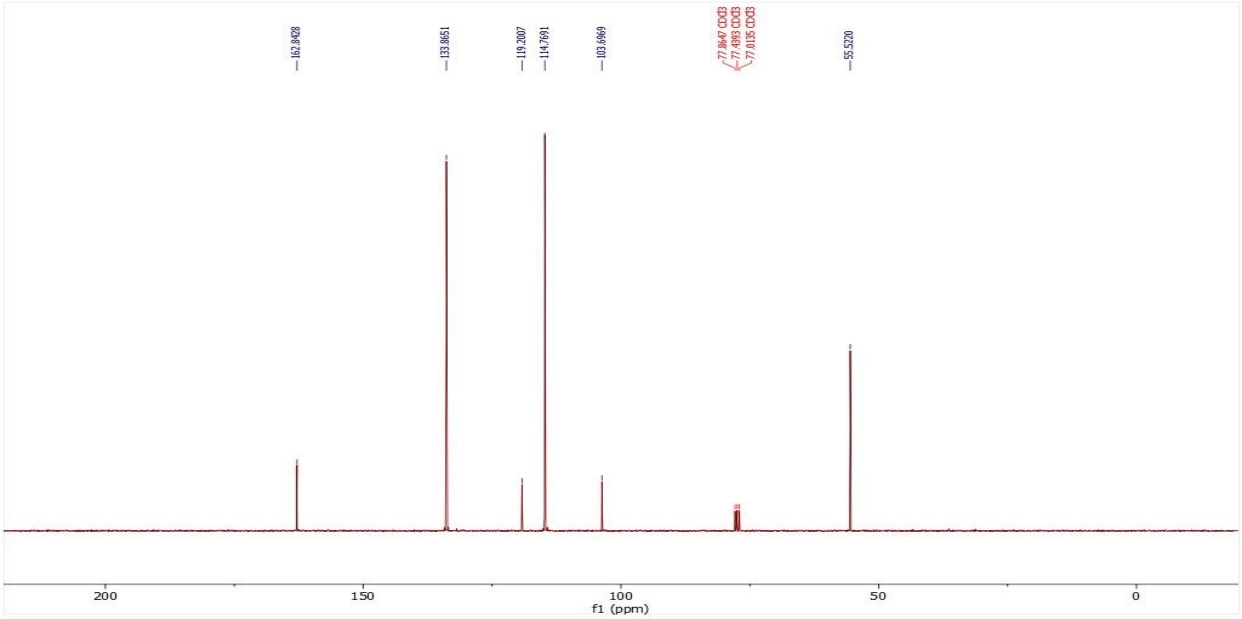

^{1}H NMR and ^{13}C NMR spectra of 4-methoxybenzonitrile

^{1}H NMR (300 MHz, $CDCl_3$): *δ* 7.60 (d, *J* = 9 Hz, 2 H), 6.97 (d, *J* = 9 Hz, 2 H), 3.88 (s, 3 H) ppm; ^{13}C NMR (75 MHz, $CDCl_3$): δ 162.8, 133.8, 119.2, 114.7, 103.6, 55.5 ppm.

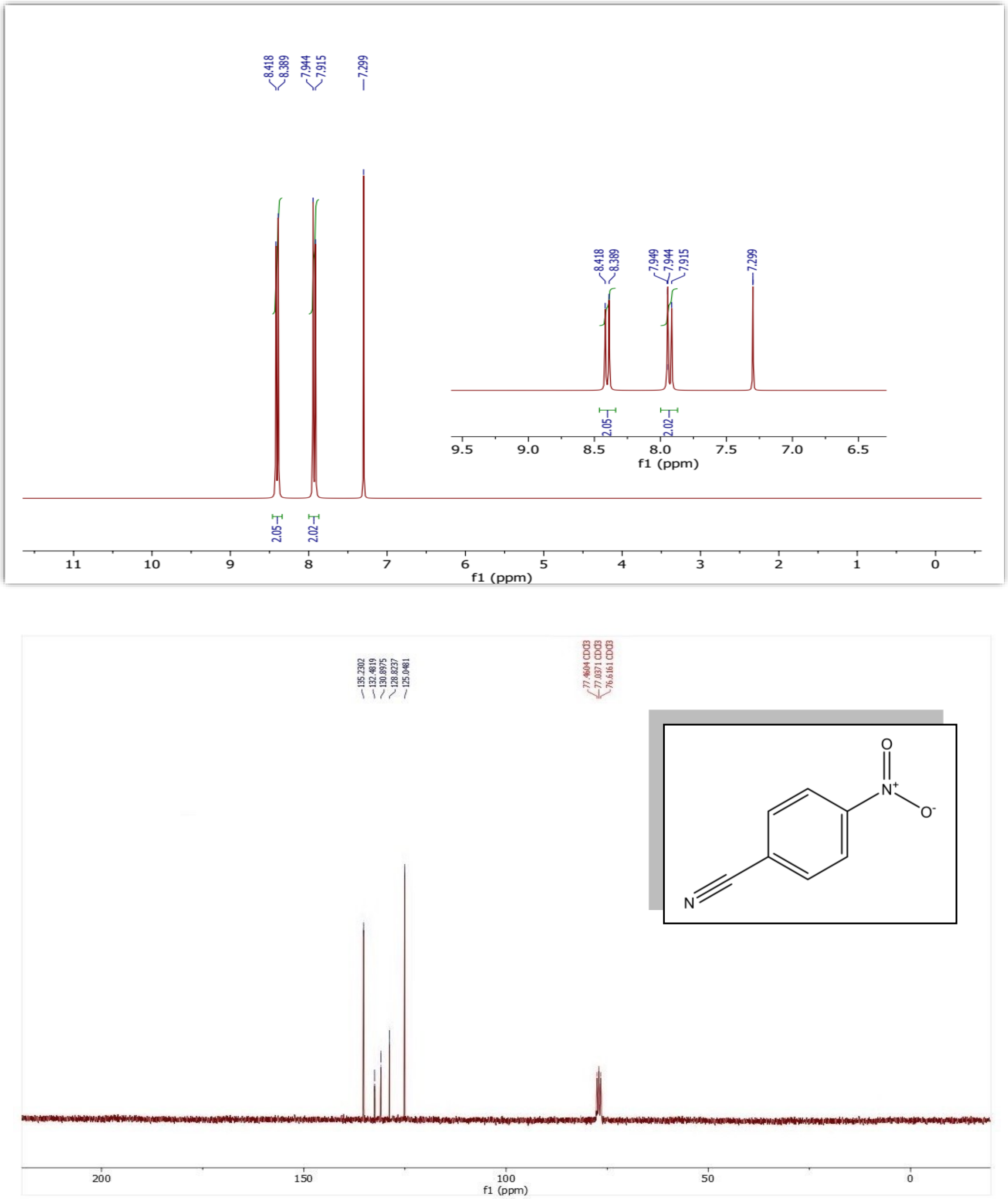

^{1}H NMR and ^{13}C NMR spectra of 4-nitrobenzonitrile

^{1}HNMR (300 MHz, $CDCl_3$): δ 8.40 (d, J = 8.7 Hz, 2 H), 7.92 (d, J = 8.7 Hz, 2 H) ppm; ^{13}C NMR (75 MHz, $CDCl_3$): δ 135.2, 132.4, 130.8, 128.8, 125.0 ppm.

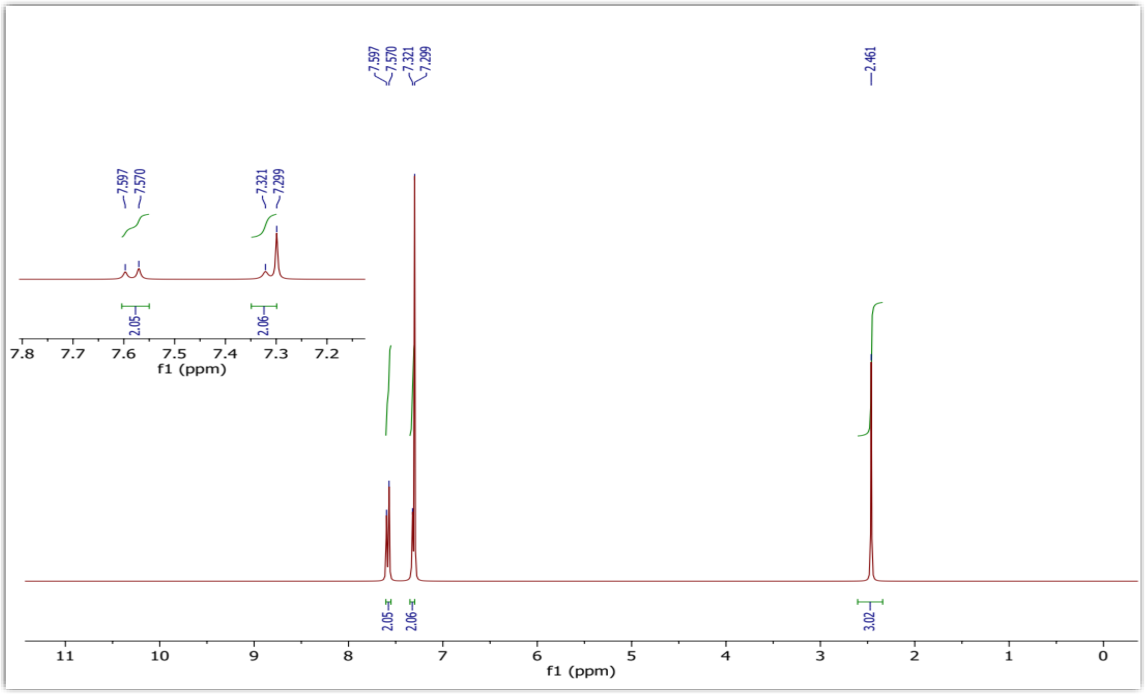

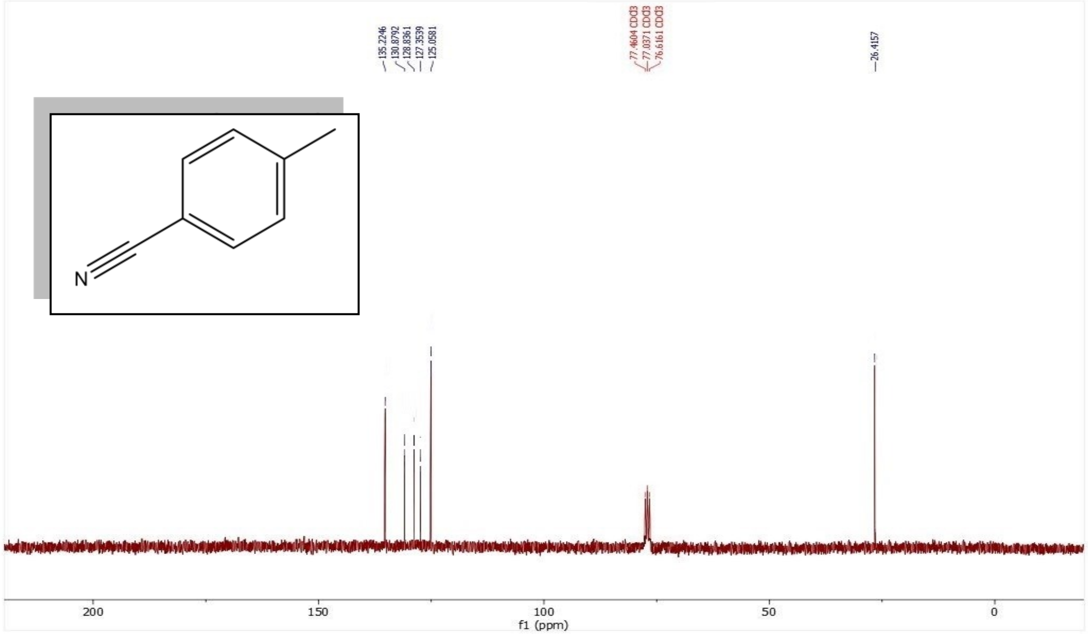

^{1}H NMR and ^{13}C NMR spectra of 4-methylbenzonitrile

^{1}H NMR (300 MHz, $CDCl_3$): δ 7.58 (d, J = 8.1 Hz, 2 H), 7.31 (d, J = 6.6 Hz, 2 H), 2.46 (s, 3 H) ppm;

^{13}C NMR (75 MHz, $CDCl_3$): δ 135.2, 130.8, 128.8, 127.3, 125.0, 26.4 ppm.

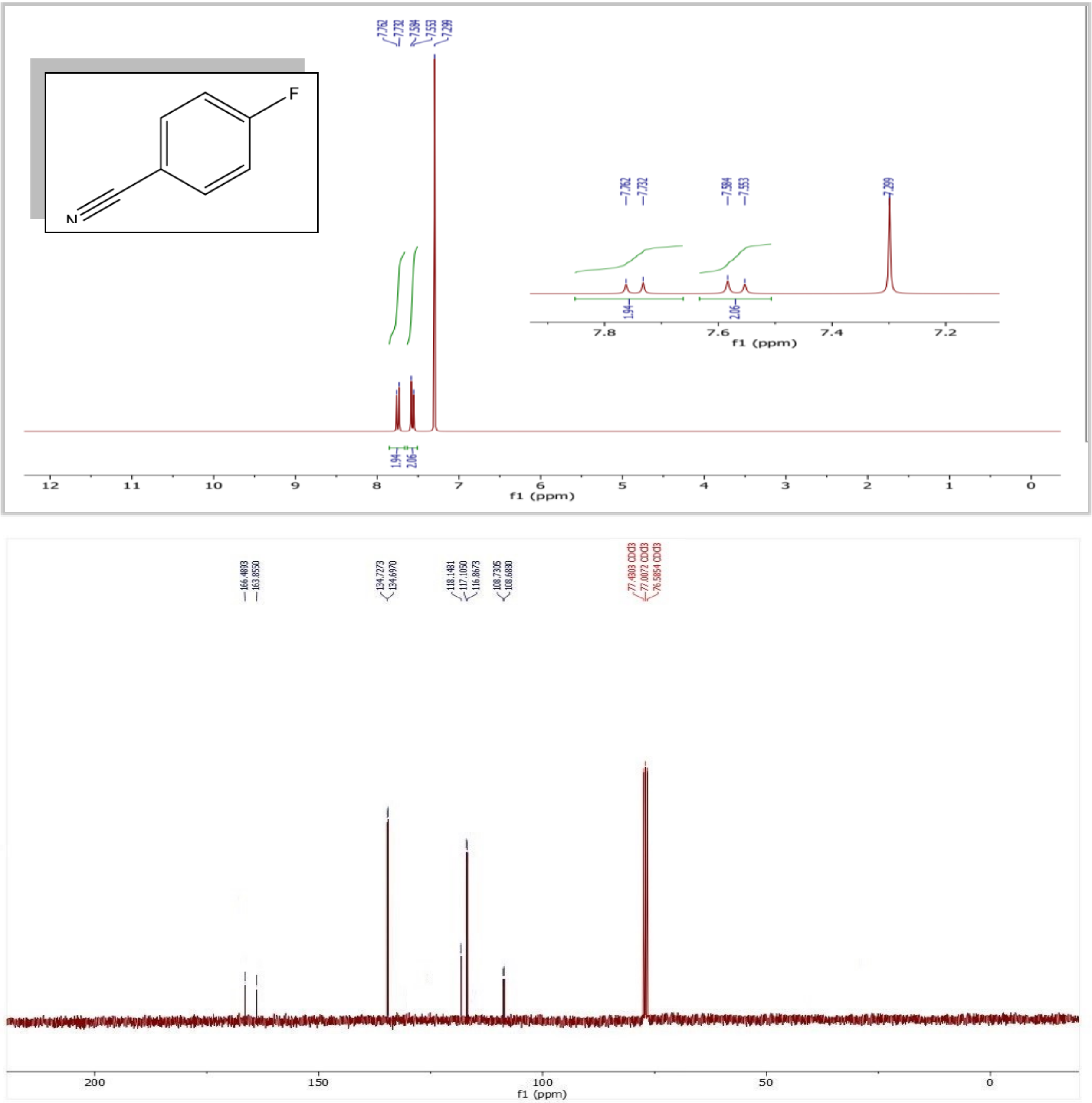

^{1}H NMR and ^{13}C NMR spectra of 4-fluorobenzonitrile

^{1}H NMR (300 MHz, $CDCl_3$): δ 7.74 (d, J = 9 Hz, 2 H), 7.56 (d, J = 9.3 Hz, 2 H) ppm; ^{13}C NMR (75 MHz, $CDCl_3$): δ 165.1 (d, $J_{C\text{-}F}$= 195 Hz), 134.6 (d, $J_{C\text{-}F}$= 7.5 Hz), 118.1, 116.9 (d, $J_{C\text{-}F}$= 22.5 Hz), 108.6 (d, $J_{C\text{-}F}$= 3.7 Hz) ppm.

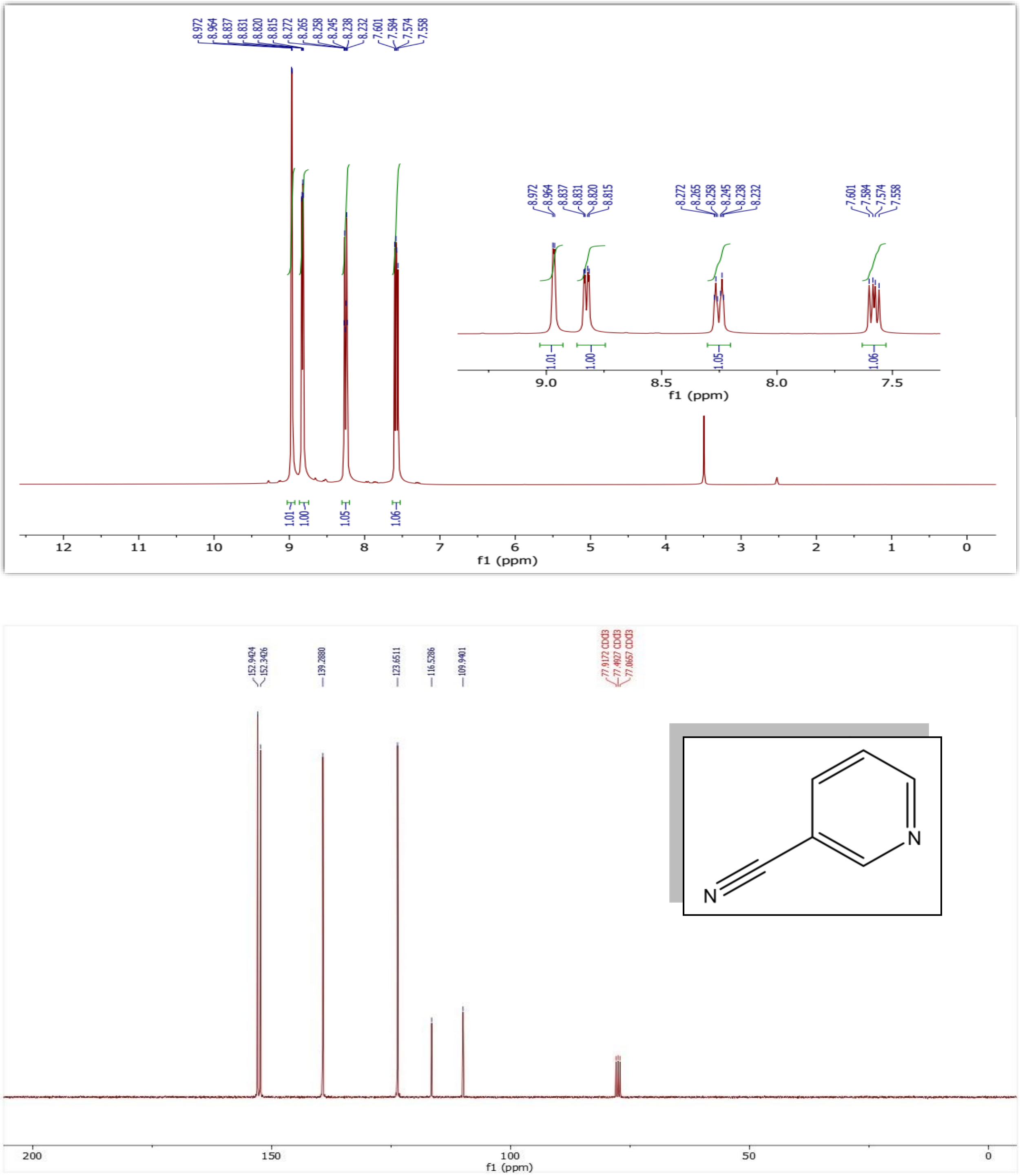

^{1}H NMR and ^{13}C NMR spectra of nicotinonitrile

^{1}H NMR (300 MHz, DMSO-d_6): δ 8.96 (s, 1 H), 8.82 (d, J = 5.1 Hz, 1 H), 8.25 (d, J = 8.1 Hz, 1 H), 7.56-7.60 (m, 1 H) ppm; ^{13}C NMR (75 MHz, $CDCl_3$): δ 152.9, 152.3, 139.2, 123.6, 116.5, 109.9 ppm.

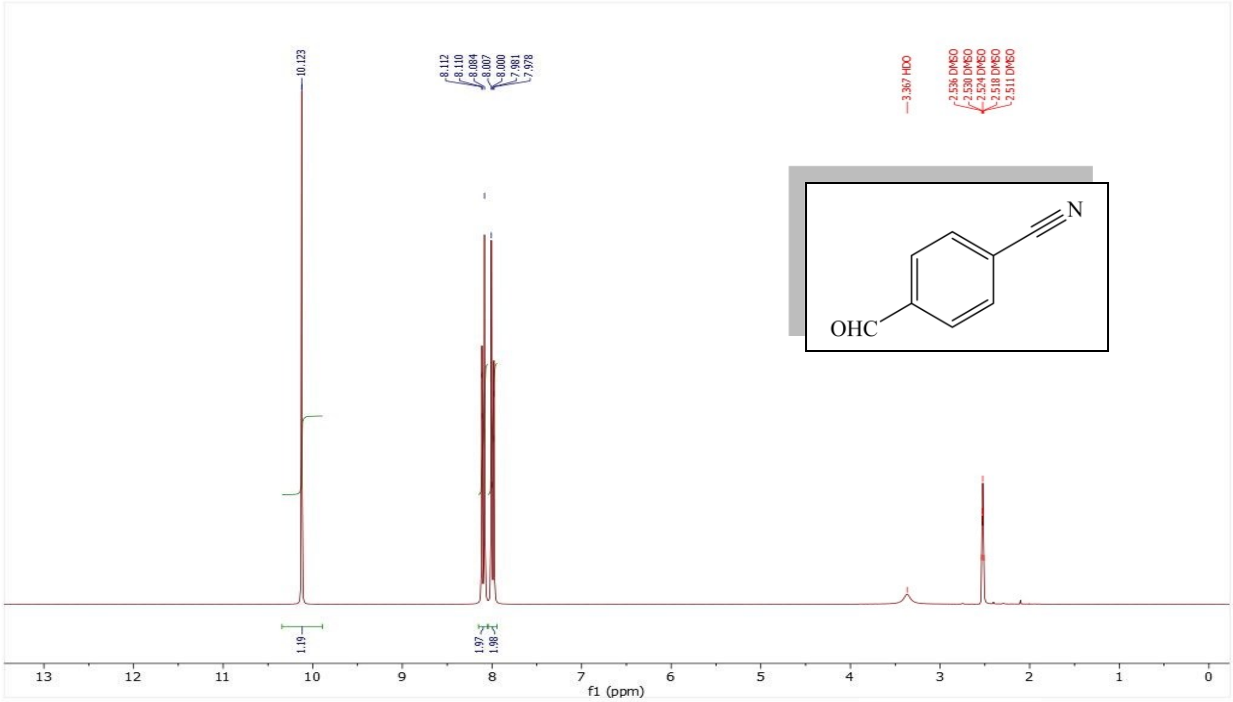

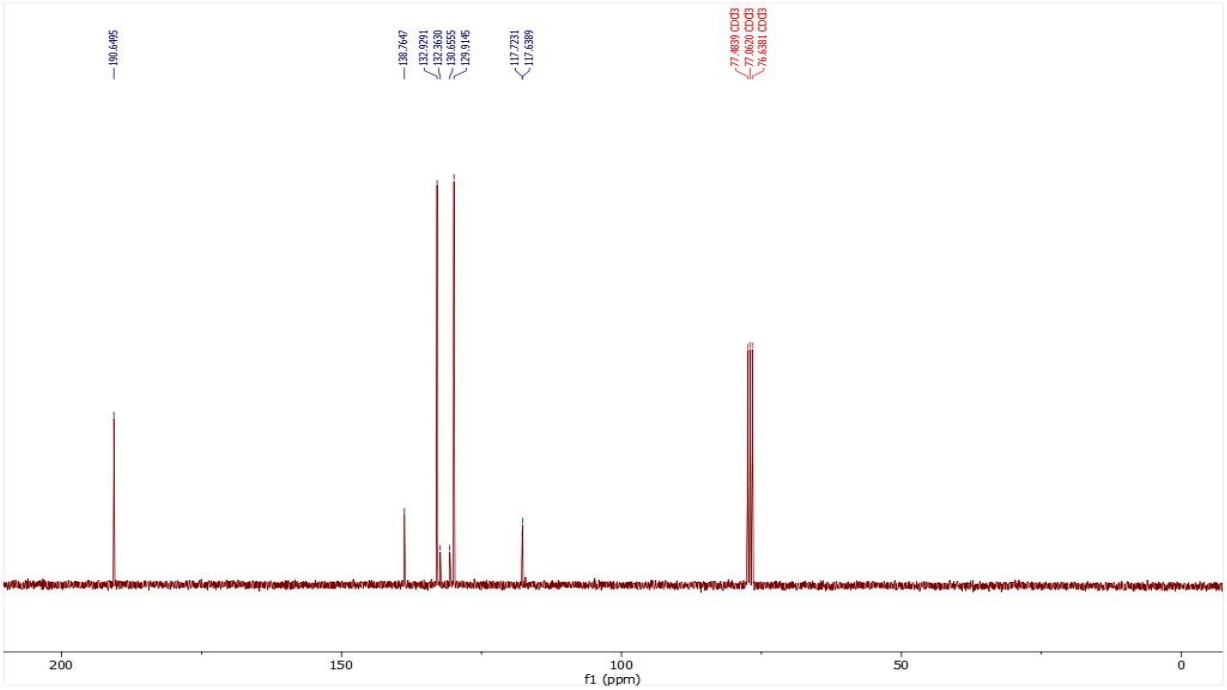

^{1}H NMR and ^{13}C NMR spectra of 4-cyanobenzaldehyde

^{1}H NMR (300 MHz, DMSO-d_6): δ 10.12 (s, 1 H), 8.09 (d, J = 7.8, 2 H), 7.99 (d, J = 7.8, 2 H) ppm; ^{13}C NMR (75 MHz, $CDCl_3$): δ 190.6, 138.7, 132.9, 129.9, 117.7 ppm.

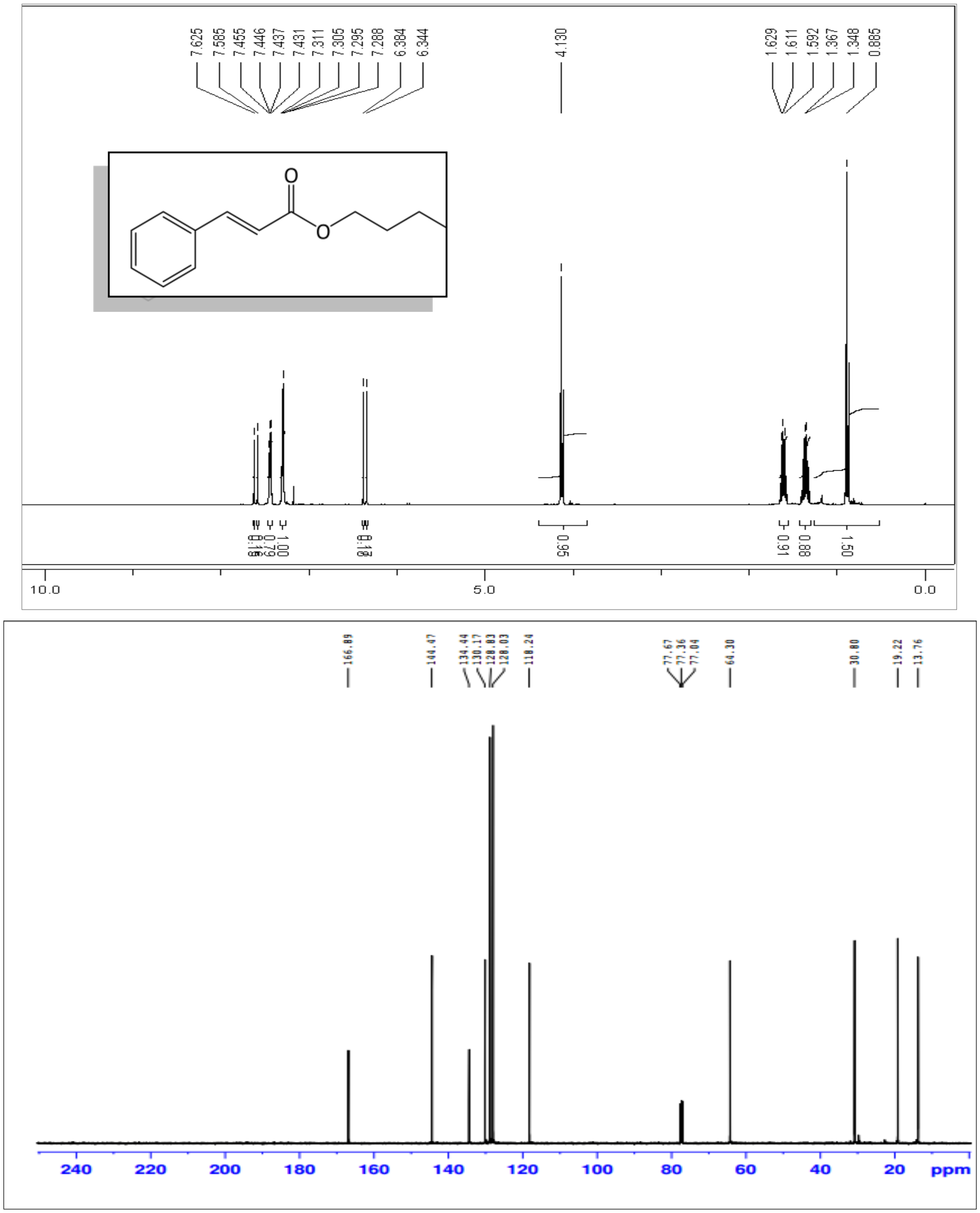

^{1}H NMR and ^{13}C NMR spectra of (E)-n-butyl cinnamate

^{1}H NMR (400 MHz, $CDCl_3$): δ 7.60 (d, 1H, ^{3}J = 16.4 Hz), 7.43-7.45 (m, 2H), 7.29-7.30 (m, 3H), 6.36 (d, 1H, ^{3}J = 16.0 Hz), 4.13 (t, 2H, ^{3}J = 6.8 Hz), 1.59-1.62 (m, 2H), 1.34-1.36 (m, 2H), δ 0.88 (t, 3H, ^{3}J = 7.6 Hz) ppm. ^{13}C NMR (100 MHz, $CDCl_3$): δ 166.8, 144.4, 134.4, 130.1, 128.8, 128.0, 118.2, 64.3, 30.8, 19.2, 13.7 ppm.

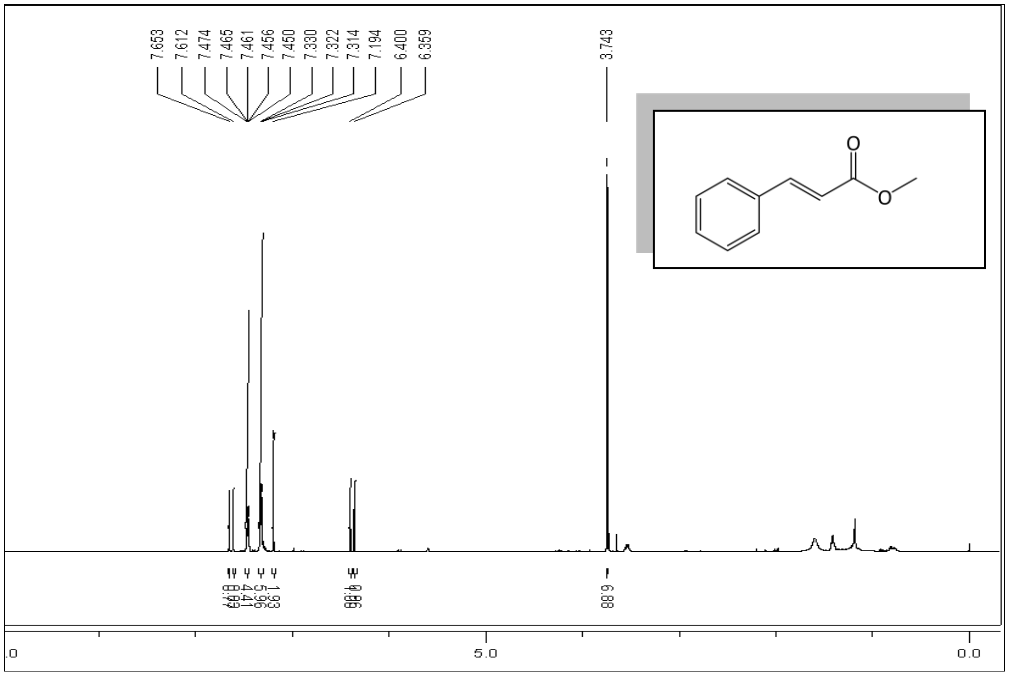

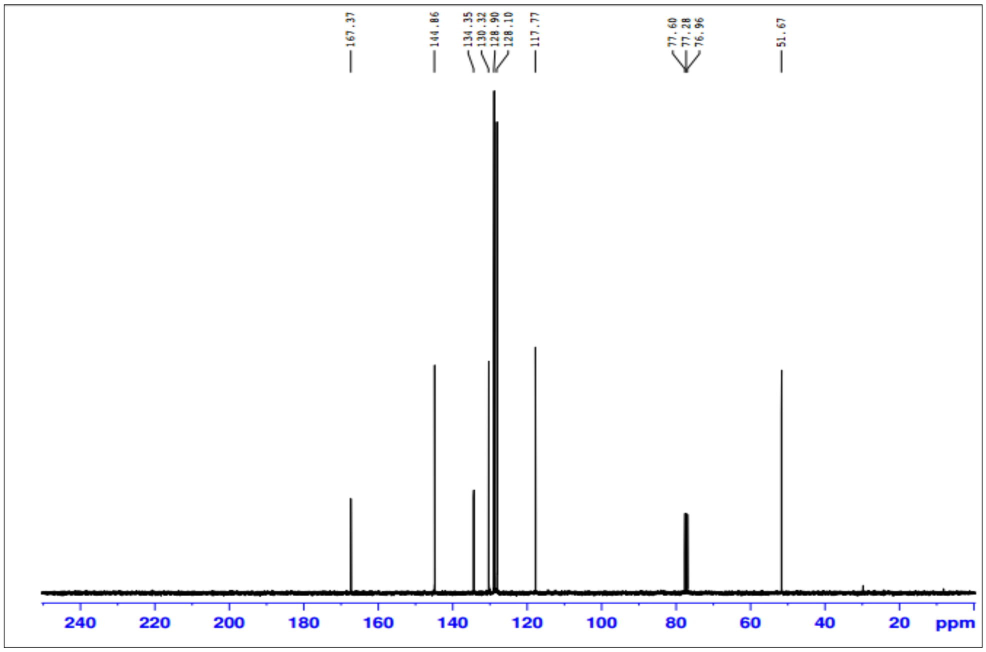

^{1}H NMR and ^{13}C NMR spectra of (E)-methyl cinnamate

^{1}H NMR (400 MHz, $CDCl_3$): δ 7.63 (d, 1H, ^{3}J = 16.4 Hz), 7.45-7.47 (m, 2H), 7.31-7.33 (m, 3H), 6.38 (d, 1H, ^{3}J = 16.4 Hz), 3.74 (s, 3H) ppm. ^{13}C NMR (100 MHz, $CDCl_3$): δ 167.3, 144.8, 134.3, 130.3, 128.9, 128.1, 117.7, 51.6 ppm.

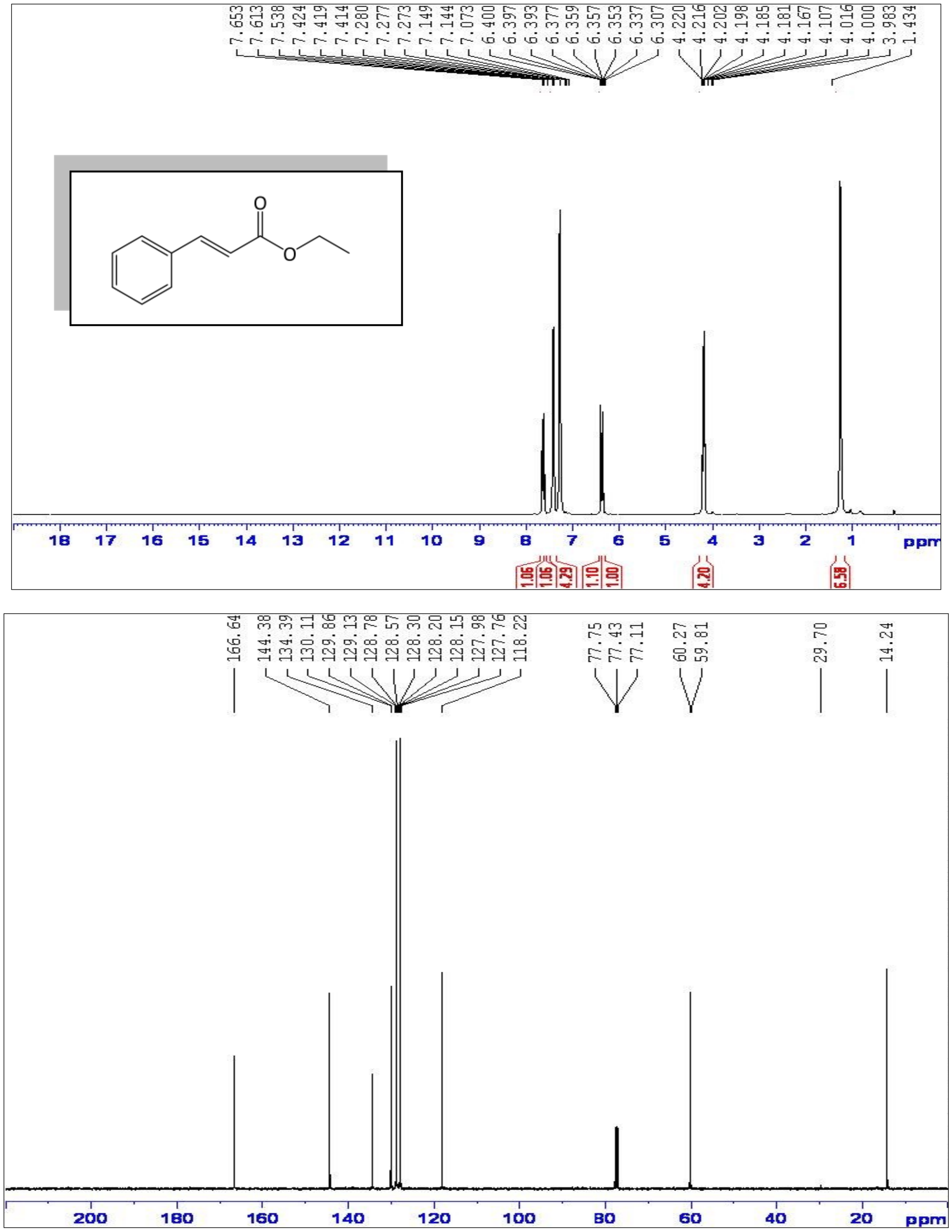

^{1}H NMR and ^{13}C NMR spectra of (E)-ethyl cinnamate

^{1}H NMR (400 MHz, $CDCl_3$): δ 7.63 (d, 1H, ^{3}J = 16.0 Hz), 7.41-7.42 (m, 2H), 7.27-7.28 (m, 3H), 6.35 (d, 1H, ^{3}J = 16.0 Hz), 4.19 (q, 2H, ^{3}J = 8.0 Hz), 1.43 (t, 3H, ^{3}J = 8.0 Hz) ppm.^{13}C NMR (100 MHz, $CDCl_3$): δ 166.6, 144.3, 134.3, 130.1, 128.7, 127.9, 118.2, 60.0, 14.2 ppm.

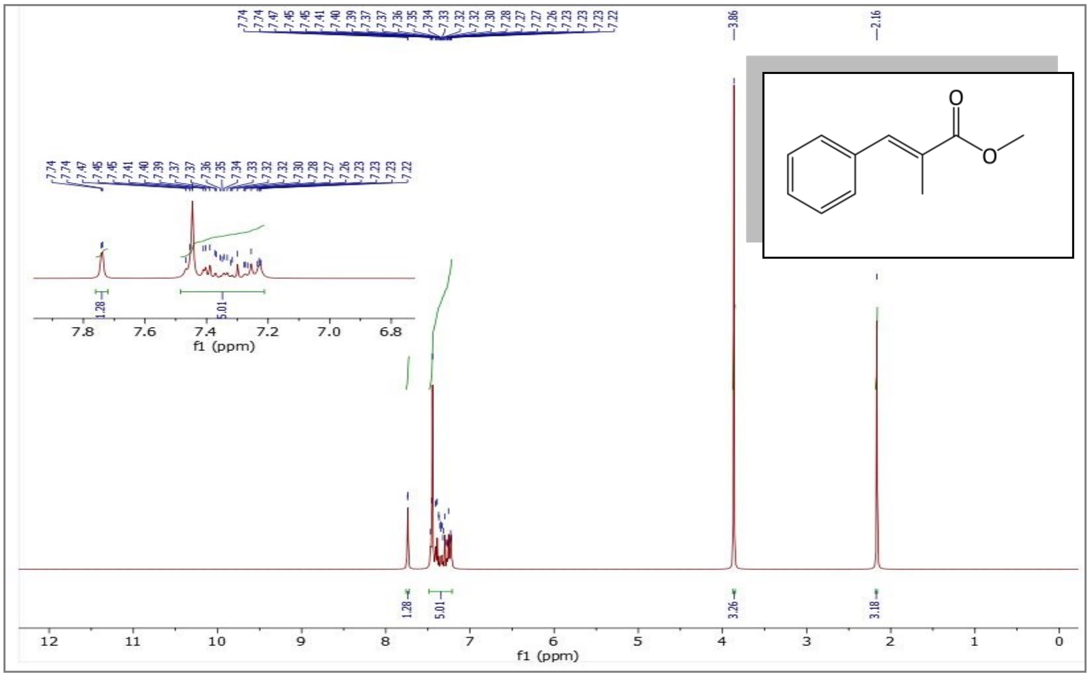

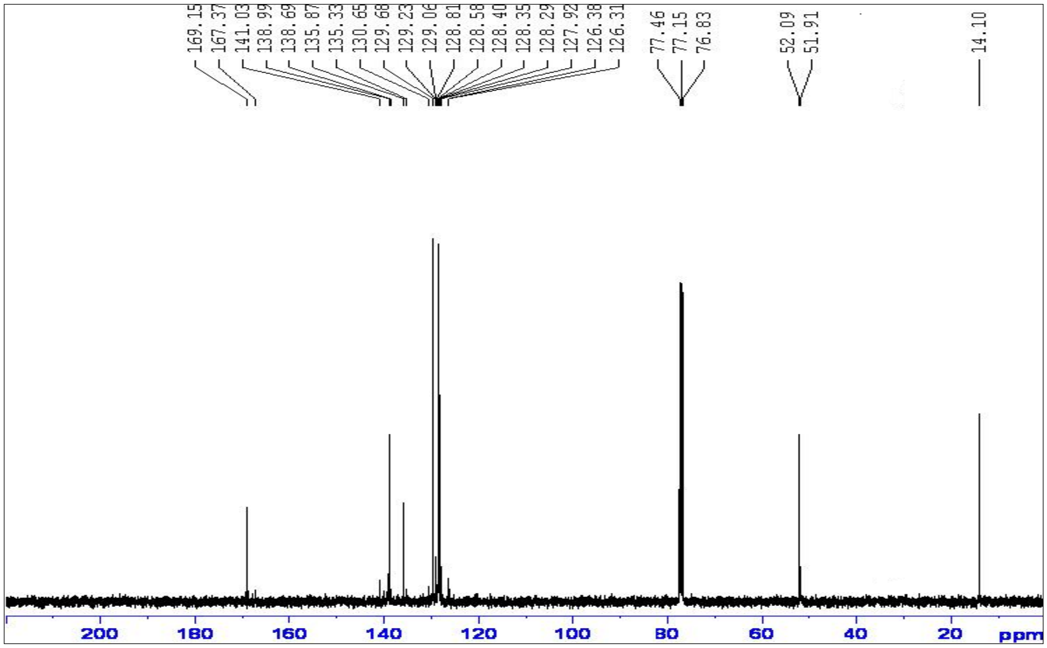

^{1}H NMR and ^{13}C NMR spectra of (E)-methyl 2-methyl-3-phenylacrylate

^{1}H NMR (300 MHz, $CDCl_3$): δ 7.74 (s, 1H), 7.22-7.47 (m, 5H), 3.86 (s, 3H), 2.16 (s, 3H) ppm.^{13}C NMR (100 MHz, $CDCl_3$): δ 169.1, 138.9, 135.8, 129.6, 127.9, 52.0, 14.1 ppm.

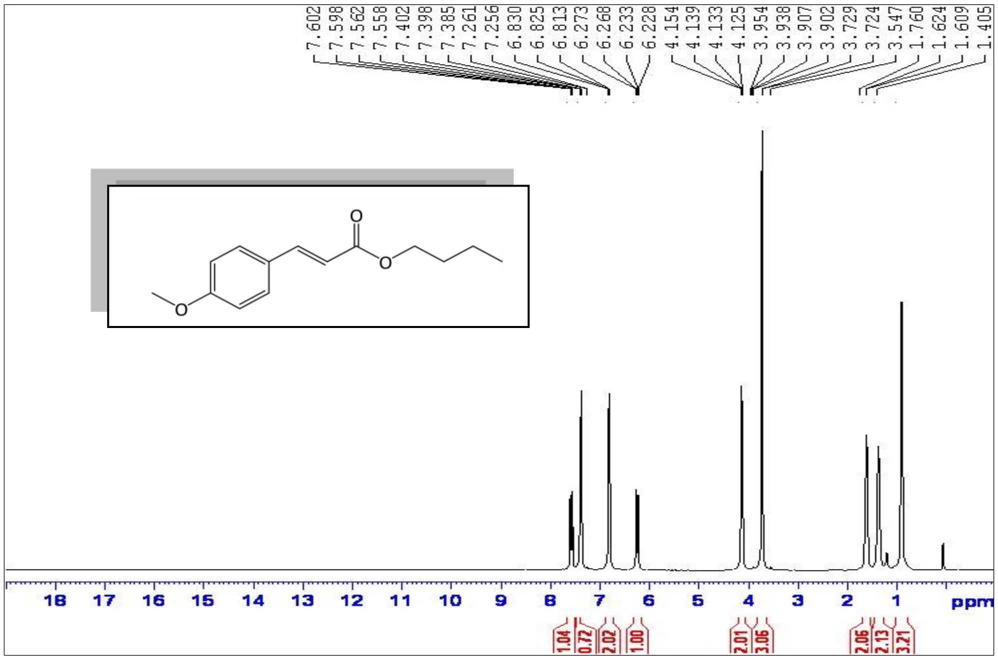

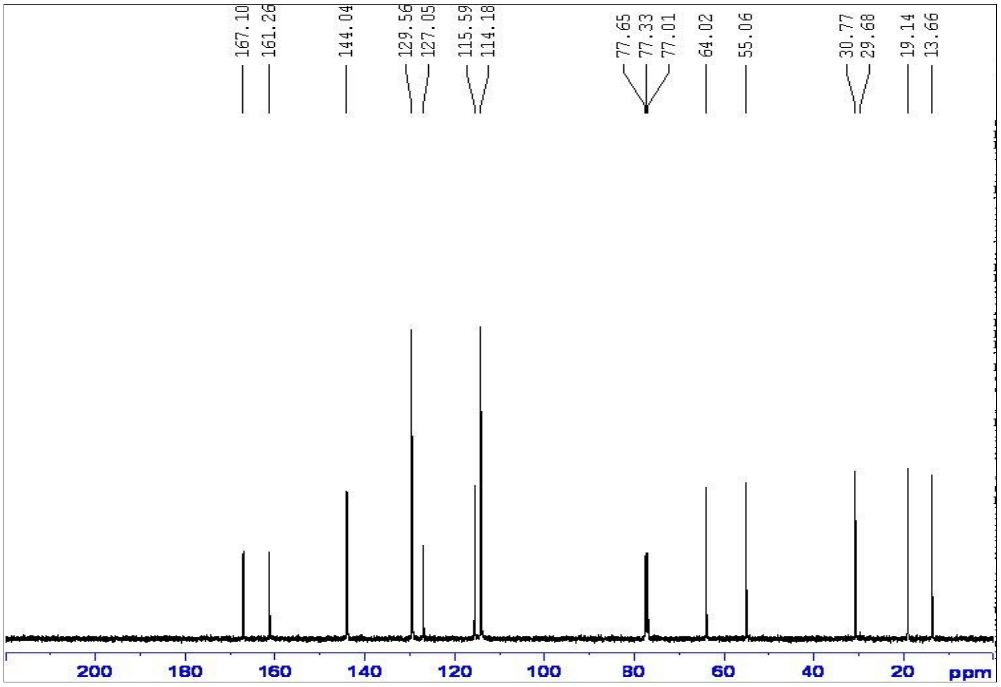

^{1}H NMR and ^{13}C NMR spectra of (E)-n-butyl 3-(4-methoxyphenyl) acrylate

^{1}H NMR (400 MHz, $CDCl_3$): δ 7.58 (d, 1H, ^{3}J = 16.0 Hz), 7.39 (d, 3H, ^{3}J =4.2 Hz), 6.24 (d, 1H, ^{3}J = 16.0 Hz), 4.13 (m, 2H, ^{3}J =7.0 Hz), 3.72 (s, 3H), 1.60-1.62 (m, 2H), 1.35-1.40 (m, 2H), 0.91 (t, 3H, ^{3}J =7.0 Hz) ppm.^{13}C NMR (100 MHz, $CDCl_3$): δ 167.1, 161.2, 144.0, 129.5, 127.0, 115.5, 114.1, 64.0, 55.0, 30.7, 19.1, 13.6 ppm.

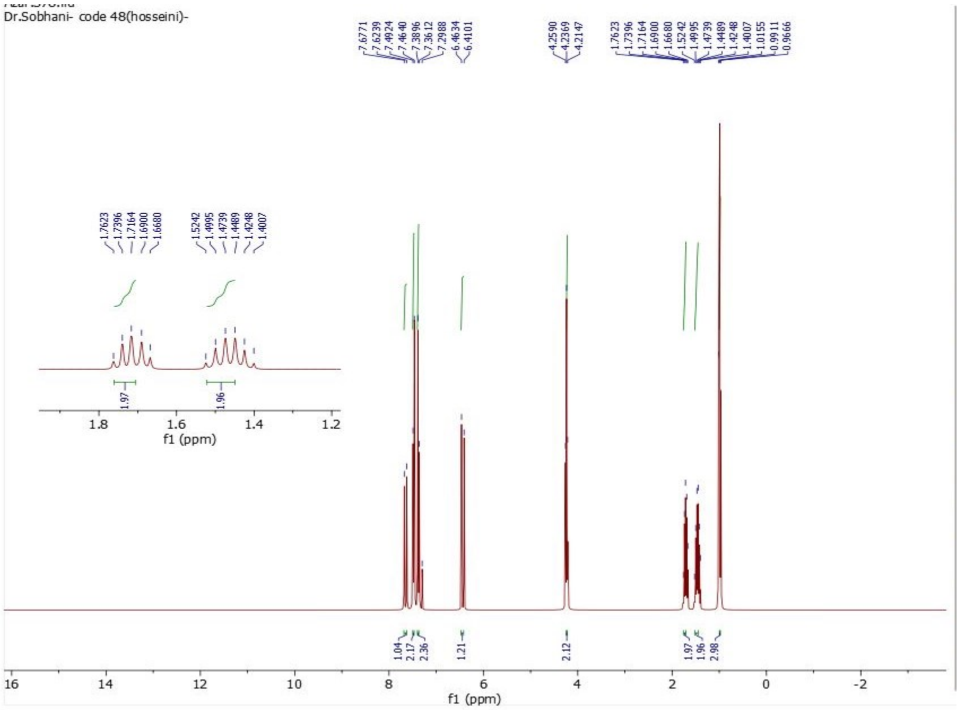

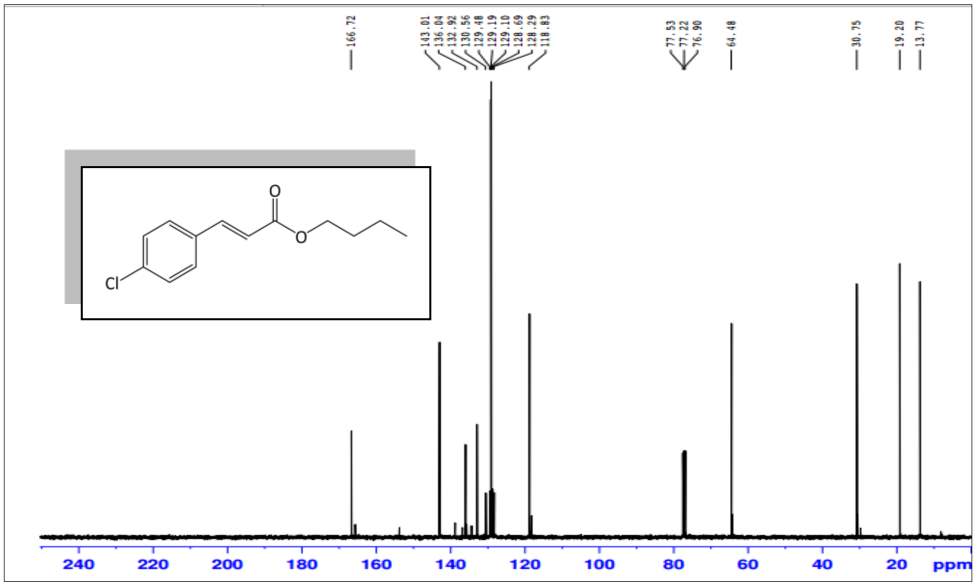

^{1}H NMR and ^{13}C NMR spectra of (E)-n-butyl 3-(4-chlorophenyl) acrylate

^{1}H NMR (300 MHz, $CDCl_3$): δ 7.65 (d, 1H, ^{3}J = 16.0 Hz), 7.47 (d, 2H, ^{3}J = 8.5 Hz), 7.37 (d, 2H, ^{3}J = 8.5 Hz), 6.43 (d, 1H, ^{3}J = 16.0 Hz), 4.24 (t, 2H, ^{3}J = 6.6 Hz), 1.40-1.52 (m, 2H), 1.67-1.76 (m, 2H), 0.99 (t, 3H, ^{3}J = 7.3 Hz) ppm.^{13}C NMR (100 MHz, $CDCl_3$): δ 166.7, 143.0, 136.0, 132.9, 129.4, 129.1, 118.8, 64.4, 30.7, 19.2, 13.7 ppm.

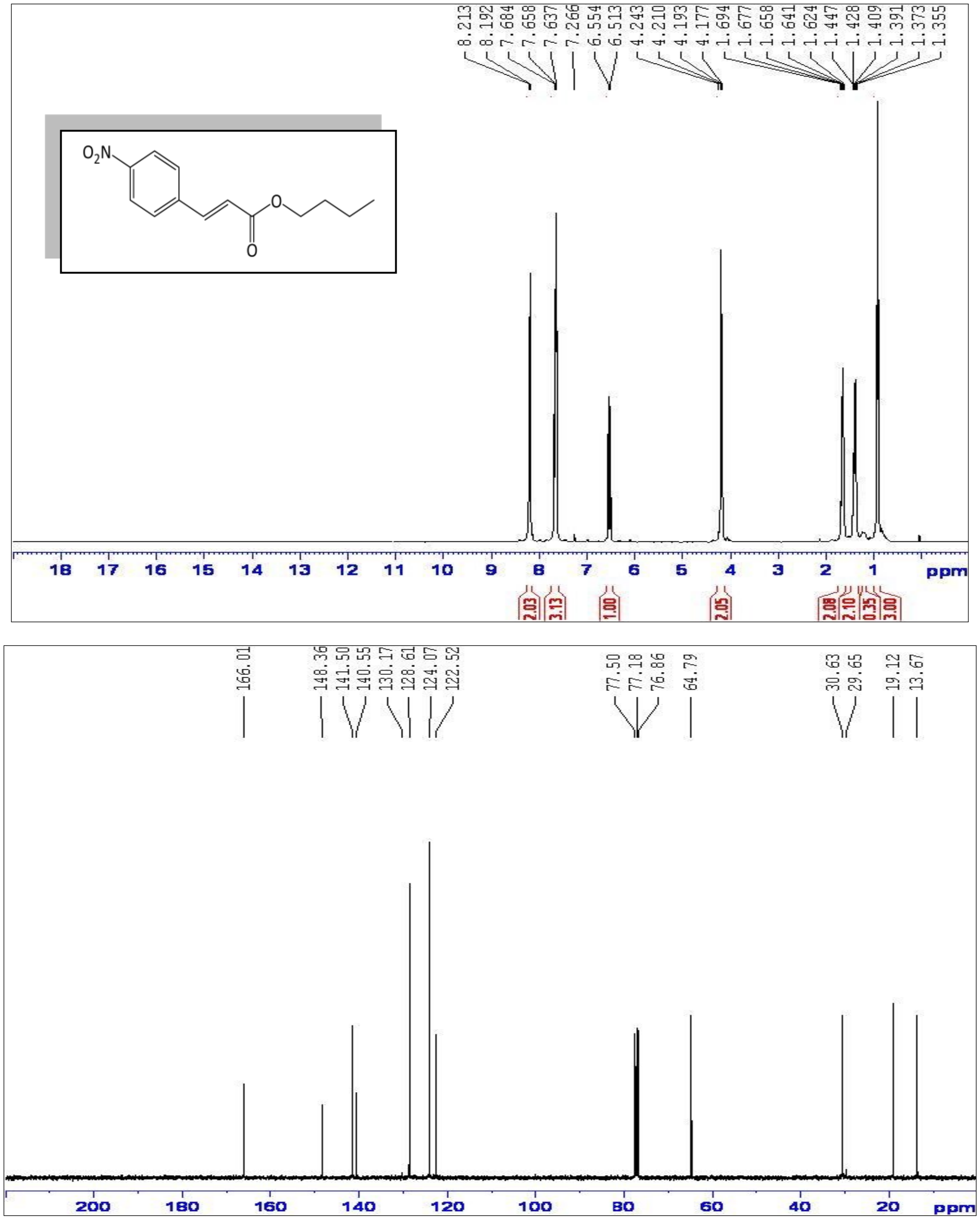

^{1}H NMR and ^{13}C NMR spectra of (E)-n-butyl 3-(4-nitrophenyl) acrylate

^{1}H NMR (400 MHz, $CDCl_3$): δ 8.20 (d, 2H, ^{3}J = 8.0 Hz), 7.63-7.68 (m, 3H), 6.53 (d, 1H, ^{3}J = 16.0 Hz), 4.17-4.24 (m, 2H), 1.62-1.69 (m, 2H), 1.35-1.44 (m, 2H), 0.92 (t, 3H, ^{3}J = 7.2 Hz) ppm. ^{13}C NMR (100 MHz, $CDCl_3$): δ 166.0, 148.3, 141.5, 140.5, 128.6, 124.0, 122.5, 64.7, 30.6, 19.1, 13.6 ppm.

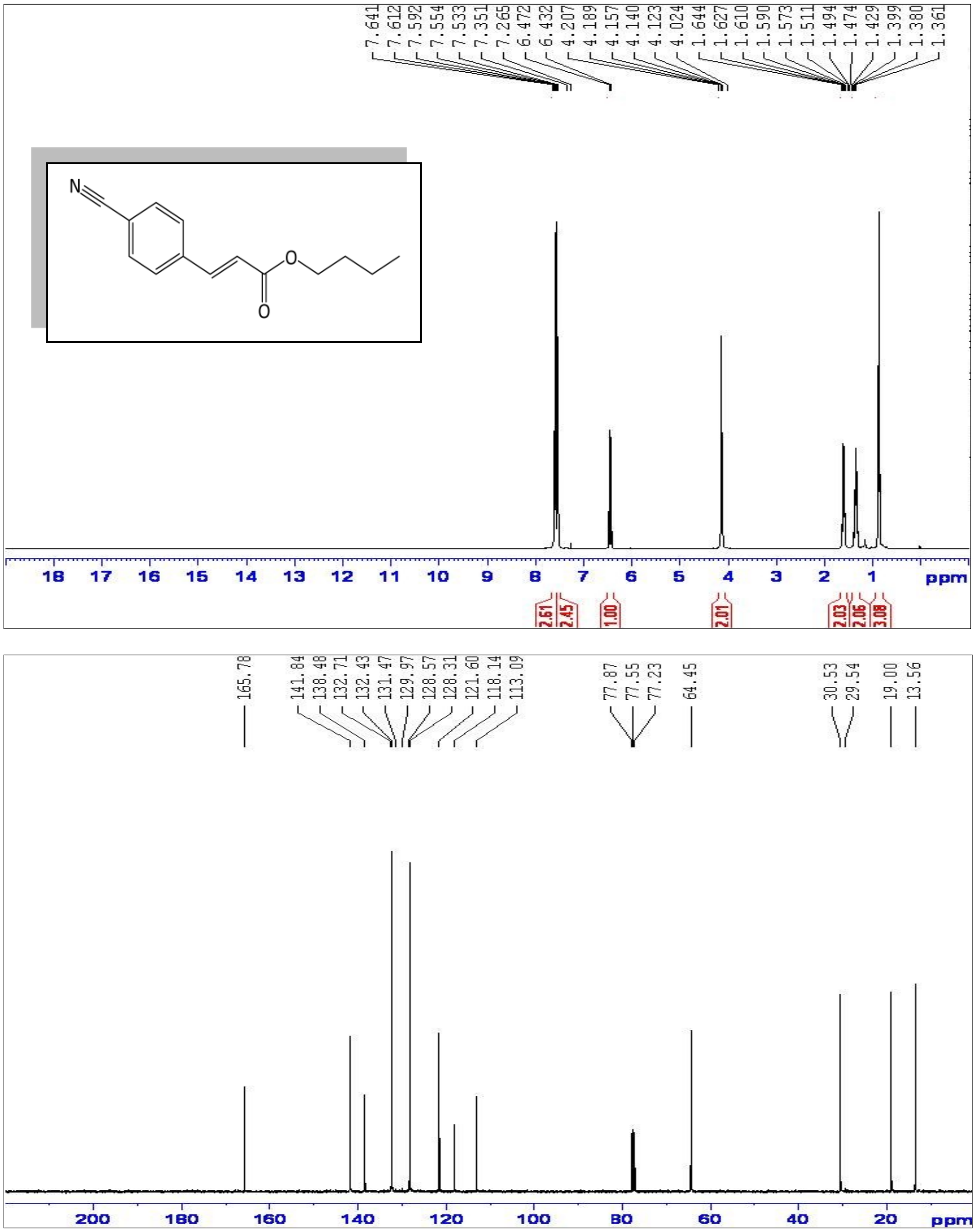

^{1}H NMR and ^{13}C NMR spectra of (E)-n-butyl 3-(4-cyanophenyl) acrylate

^{1}H NMR (400 MHz, $CDCl_3$): δ 7.60 (d, 2H, ^3J = 8.0 Hz), 7.57 (d, 1H, ^3J = 15.2 Hz), 7.54 (d, 2H, ^3J = 8.4 Hz), 6.45 (d, 1H, ^3J = 16.0 Hz), 4.14 (t, 2H, ^3J = 6.8 Hz), 1.57-1.64 (m, 2H), 1.30-1.38 (m, 2H), 0.77 (t, 3H, ^3J = 7.0 Hz) ppm. ^{13}C NMR (100 MHz, $CDCl_3$): δ 165.7, 141.8, 138.4, 132.4, 128.3, 121.6, 118.1, 113.0, 64.4, 30.5, 19.0, 13.5 ppm.
